# Novel insights and an updated review of metabolic syndrome in immune-mediated organ transplant rejection

**DOI:** 10.3389/fimmu.2025.1580369

**Published:** 2025-04-22

**Authors:** Zetong Tao, Zilong Luo, Zifeng Zou, Weicong Ye, Yanglin Hao, Xiaohan Li, Kexiao Zheng, Jie Wu, Jiahong Xia, Yang Zhao, Yongjun Wang, Xi Zhang

**Affiliations:** Department of Cardiovascular Surgery, Union Hospital, Tongji Medical College, Huazhong University of Science and Technology, Wuhan, China

**Keywords:** metabolic syndrome, organ transplantation, transplant rejection, immune system, metabolites and immune cell

## Abstract

Metabolic syndrome (MetS) is a group of symptoms that are characterized by abnormal changes in metabolic substances such as glucose, lipids, proteins, and bile acids. MetS is a common complication after organ transplantation and can further affect the survival and physiological function of the graft by reprograming the patient’s immune environment. Additionally, MetS can influence the occurrence of post-transplant complications, such as infections. In recent years, research into the epidemiology and mechanisms of MetS has grown significantly. In this review, we summarize the mechanisms of MetS after transplantation and the mechanisms of hyperglycemia, insulin resistance, hyperlipidemia, abnormal bile acids, and abnormal amino acids on the body’s immune cells as related to the effect of metabolic disorders on immune rejection after liver, kidney, heart, skin and other organ transplantation. Finally, we provide an overview of current treatment strategies and offer insights into potential future therapies for managing MetS in transplant recipients.

## 1 Introduction

**Figure 1:**
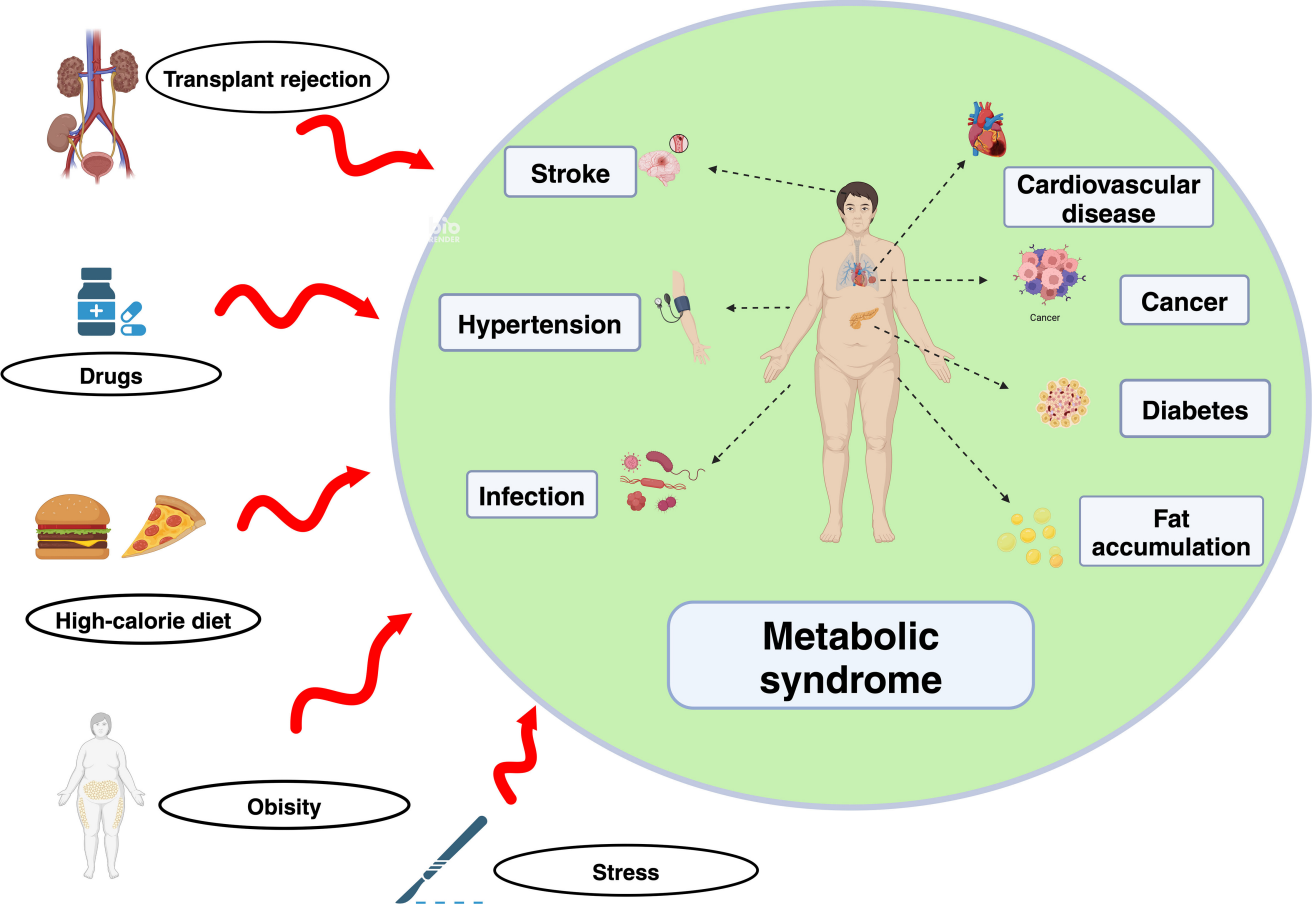
Etiology and adverse effects of MetS. Obesity caused by a high-sugar and high-fat diet is the cause of metabolic syndrome in most people, and the application of some drugs, such as steroid hormones, can also lead to drug-induced MetS. Stress after surgery and postoperative nutritional supplementation are also important causes of MetS. Organ transplant recipients are at a high risk of MetS due to drug and lifestyle changes after surgery, which results in a high incidence of MetS. These metabolic abnormalities are more likely to lead to cardiovascular and cerebrovascular accidents and infections and tumors caused by immune disorders.

Metabolic syndrome (MetS) was first proposed in 1988 and is characterized by dyslipidemia, hypertension, and insulin resistance (IR) in a hypertriglyceride-low HDL-cholesterol state; with the development of this research, the scope of metabolic abnormalities has gradually increased (1). Organ transplantation is still the best method for the treatment of end-stage organ failure. In recent years, due to improvements in surgical techniques and postoperative management, the survival rate of transplant patients has significantly increased (2). However, metabolic abnormalities, such as blood glucose, blood lipids, and amino acids, that often occur after transplantation strongly affect the survival of grafts and the quality of life of patients. Among the latest diagnostic indicators of metabolic syndrome, waist circumference is usually used as an indicator of abdominal obesity to establish a rough estimation. Comprehensive evaluations of fasting serum triglyceride, high-density lipoprotein, and cholesterol levels; blood pressure; fasting blood glucose; and other indicators are needed to further determine risks (1, 3). Current studies have shown that stress caused by transplantation surgery, drug and lifestyle changes after transplantation, and a series of metabolic abnormalities that occur before and after transplantation have regulatory effects on the immune system, which alters the normal immune environment in different ways and affects the immune tolerance and tolerance of the graft. The result is reduced graft survival time, cardiovascular disease risk, infection, cancer, hypertension and other complications (**Figure 1**) (3).

In this review, we summarize the current impacts of MetS on transplant immune rejection. Starting with the pathophysiological mechanism of MetS, we first discuss the impact of the abnormal metabolism of lipids, sugars, bile acids, and amino acids in MetS on immune cells and immune signaling pathways in the immune system. Then, we discuss the relationship between organ transplant rejection and MetS. Finally, current management strategies and prospects for transplantation are discussed.

## 2 Pathophysiological mechanisms of MetS

### 2.1 Mechanisms of insulin resistance

Insulin is a peptide hormone secreted by pancreatic beta cells in response to elevated blood glucose. Its main role is to promote the ability of effector cells to store glucose as glycogen in the liver and skeletal muscle and to convert glucose into fatty acids and lipids, ultimately resulting in decreased blood glucose (4). As a result of surgical stress and the side effects of immunosuppressive drugs, the reduced insulin sensitivity and insulin secretion of effector cells often leads to insulin transplantation resistance and posttransplant diabetes (5). When insulin resistance occurs in skeletal muscle, the inhibition of insulin-mediated lipolysis is reduced, the oxidative breakdown of fatty acids is increased, and the increase in free fatty acids (FFAs) further inhibits the function of PI3 kinase and GLUT-4 in the insulin transport pathway and exacerbates insulin resistance (6–8). Increased FFAs also cause cholesterol and triglyceride (TG) levels to increase, which further causes TG levels to shift from vLDL to HDL and reduces HDL concentrations (9). Abnormal blood lipid changes can lead to the activation of macrophages and the deposition of cholesterol in blood vessels, which leads to pathological changes in atherosclerosis in MetS patients (10). Moreover, an increase in FFAs leads to a decrease in brachial artery dilatation function and hypertension in MetS patients (11); additionally, insulin resistance in MetS patients increases blood viscosity (12).

### 2.2 Secretory mechanisms in adipose tissue

Adipose tissue is a tissue with endocrine properties and its dysfunction is one of the main causes and pathogenic mechanisms of MetS. A variety of cytokines and hormones, such as TNF-α, IL-6, and IL-8, as well as plasmid proactivator inhibitor-1, angiotensin-II, adiponectin, and leptin, are secreted (13).

Generally, adipose tissue plays a key role in MetS. The role of leptin is related to the inhibition of food intake and increasing energy expenditure (14) and it has also been linked to the activation of immune function and cardiovascular and cerebrovascular diseases (15, 16). In chronic hyperleptinemia and leptin resistance, continuous stimulation of the sympathetic nerve by leptin leads to vasospasm and hypertension, and leptin can further change sympathetic nerve excitability by regulating renal Na excretion. Additionally, in chronic hyperleptinemia, it can indirectly influence the occurrence of heart disease by enhancing platelet aggregation and fibrinolytic injury and promote angiogenesis (16) and it has become a key molecule linking obesity, MetS and cardiovascular injury. Adiponectin is another important molecule that is secreted by adipose tissue and plays important antiapoptotic, insulin sensitivity, anti-inflammatory and antifibrotic roles (17). Some studies have shown that its deletion may lead to MetS in mice (18). Another adipokine secreted by adipocytes, chemerin, has been implicated in inflammation, adipogenesis, angiogenesis, and energy metabolism in individuals with MetS (19). Small cohort studies have shown that the level of chemerin is greater in individuals with *de novo* MetS (20–22), which suggests that chemerin may be involved in the development of MetS.

### 2.3 Other relevant molecular mechanisms

As a widely studied cytokine, IL-6 can be secreted by a variety of cells, such as adipocytes and lymphocytes, in MetS (23, 24). Studies have shown that IL-6 has a steroid-like effect, which can affect lipid metabolism by affecting macrophage recruitment, improving insulin resistance (25), and enhancing adrenal function (26). Moreover, it also acts on the circulatory system and can affect mitochondrial function, which can lead to atherosclerosis (27). TNF-α can be secreted by adipose tissue (23), and macrophages that accumulate in adipose tissue are an important source of TNF-α release (28). The increased release of TNF-α (29), which can affect insulin action through the NF-κB, IKK-β and JNK systems (30, 31), serine phosphorylation, and loss of downstream receptor molecules (32), has been reported in obese and insulin-resistant patients. The proinflammatory effects of TNF-α on the arterial wall suggest that it also plays a key role in atherosclerosis (31). Moreover, with increasing intracellular cAmp, intracellular fatty acid breakdown and the concentration of FFAs increase (33).

As a secreted protein, Fetuin-A is involved in a variety of metabolic processes and its association with MetS has attracted increasing attention (34). Increased secretion of Fetuin-A has been observed in adipose tissue and the viscera of obese patients (35, 36)and has been shown to induce insulin resistance by interfering with GLUT-4 and activating Akt (37).

### 2.4 Mechanisms of gut microbiota metabolism

In recent years, the mechanisms underlying the relationship between gut microbiota and the development of MetS have garnered significant attention. Gut microbiota can induce and exacerbate MetS through multiple pathways, including influencing nutrient absorption, promoting chronic inflammation, and disrupting bile acid homeostasis. The balance between pathogenic and beneficial microbiota is fundamental to maintaining metabolic stability; however, this equilibrium is often disrupted in MetS (38–40). One of the consequences of dysbiosis in gut microbiota is chronic inflammation. Under the combined effects of external factors and inflammation, the integrity of the intestinal barrier is compromised, allowing endotoxins produced by pathogenic bacteria to enter the systemic circulation more easily, thereby inducing low-grade systemic inflammation (41, 42). When external factors lead to gut microbiota dysbiosis, the expression of LPS increases, driving Toll-like receptor (TLR) signaling, degrading the mucosal layer, and triggering endotoxemia. This process promotes the production of trimethylamine (TMA), a pro-atherogenic metabolite, ultimately contributing to metabolic dysregulation (43, 44).

Bile acids, as endocrine signaling molecules, exert critical regulatory roles in glucose and lipid metabolism through nuclear farnesoid X receptor (FXR) and membrane-bound G protein-coupled receptor 5 (TGR5). Beyond facilitating the absorption of lipid-soluble nutrients, they orchestrate multiple metabolic processes including glucose homeostasis, lipid regulation, and energy balance. Dysregulation of bile acid metabolism significantly contributes to the pathogenesis and progression of MetS (45). Bile acid biosynthesis is mediated via two primary enzymatic pathways: cholesterol 7α-hydroxylase (CYP7A1) and sterol 27-hydroxylase (CYP27A1), whose expression is modulated by microbial activity (46, 47). Emerging evidence indicates that gut microbial communities enriched in bile salt hydrolase (BSH)-producing species hydrolyze conjugated bile acids into diverse secondary forms, thereby establishing an expanded bile acid pool with enhanced functional complexity (48, 49).

Dietary interventions modulate gut microbial composition, subsequently reshaping bile acid metabolism. Specific microbiota subpopulations eliminate endogenous FXR antagonists such as tauro-β-muricholic acid (TβMCA), thereby potentiating FXR signaling. Concurrently, these microbial consortia generate secondary bile acids (e.g., deoxycholic acid and lithocholic acid) that act as potent ligands for TGR5 (50). Mechanistically, TGR5 activation enhances energy expenditure via thermogenesis in brown adipose tissue and skeletal muscle, while concurrently stimulating glucagon-like peptide-1 (GLP-1) secretion from intestinal L cells. Notably, L cells co-express FXR, which transcriptionally regulates GLP-1 synthesis, establishing a synergistic crosstalk between FXR and TGR5 signaling in metabolic regulation (47, 51).

### 2.5 Etiology and mechanisms of MetS after transplantation

The main cause of dyslipidemia after transplantation is the use of immunosuppressive drugs; for example, the use of corticosteroids can lead to hyperlipidemia through increased appetite, insulin resistance, and increased synthesis of triglycerides and cholesterol. Calcineurin inhibitors cause hyperlipidemia by reducing bile acid excretion, inhibiting insulin secretion and increasing FFAs. The side effects of mTOR inhibitors on the development of hyperlipidemia are greater than those of calcineurin inhibitors. The application of mTOR inhibitors mainly leads to an increase in triglycerides and leads to an increase in FFAs, which is associated with the inhibition of LPL, decreased catabolism of apolipoprotein B-100-containing lipoproteins, and excessive production of VLDL-C (52–54).

The incidence of post-transplantation diabetes mellitus (PTDM) is associated with obesity, insulin resistance and race. Specifically, certain races may be more sensitive to immunosuppressive agents. Older age, higher blood lipid levels, and an inflammatory state are also important factors for the development of PTDM in African American patients (55). The immunosuppressive drugs that are calcineurin inhibitors, cyclosporine and tacrolimus, can also cause PTDM through glucose transporter 4 (GLUT4), changes to glucokinase function that induces β-cell apoptosis, and blockage of the nuclear factor of activated T cells (NFATc) and other mechanisms (2). The contribution of tacrolimus to the development of insulin resistance is relatively high because it binds to the high level of FK506-binding protein in pancreatic β cells and inhibits insulin secretion (53). Steroids decrease the sensitivity of the liver to insulin, which leads to increased gluconeogenesis so that even when the pancreas releases insulin, the liver continues to release glucose, leading to increased appetite, fluid retention, and weight gain (56). Genetic studies have shown that peroxisome proliferator-activated receptor α (PPARα) is an important regulator of glucose and lipid metabolism and donor PPARα gene polymorphisms affect susceptibility to metabolic disorders after liver transplantation (57). A persistent graft–host immune response and immunosuppression-mediated infection, which induce islet β-cell apoptosis, are also involved in the pathogenesis of PTDM (58, 59).

**Figure 2:**
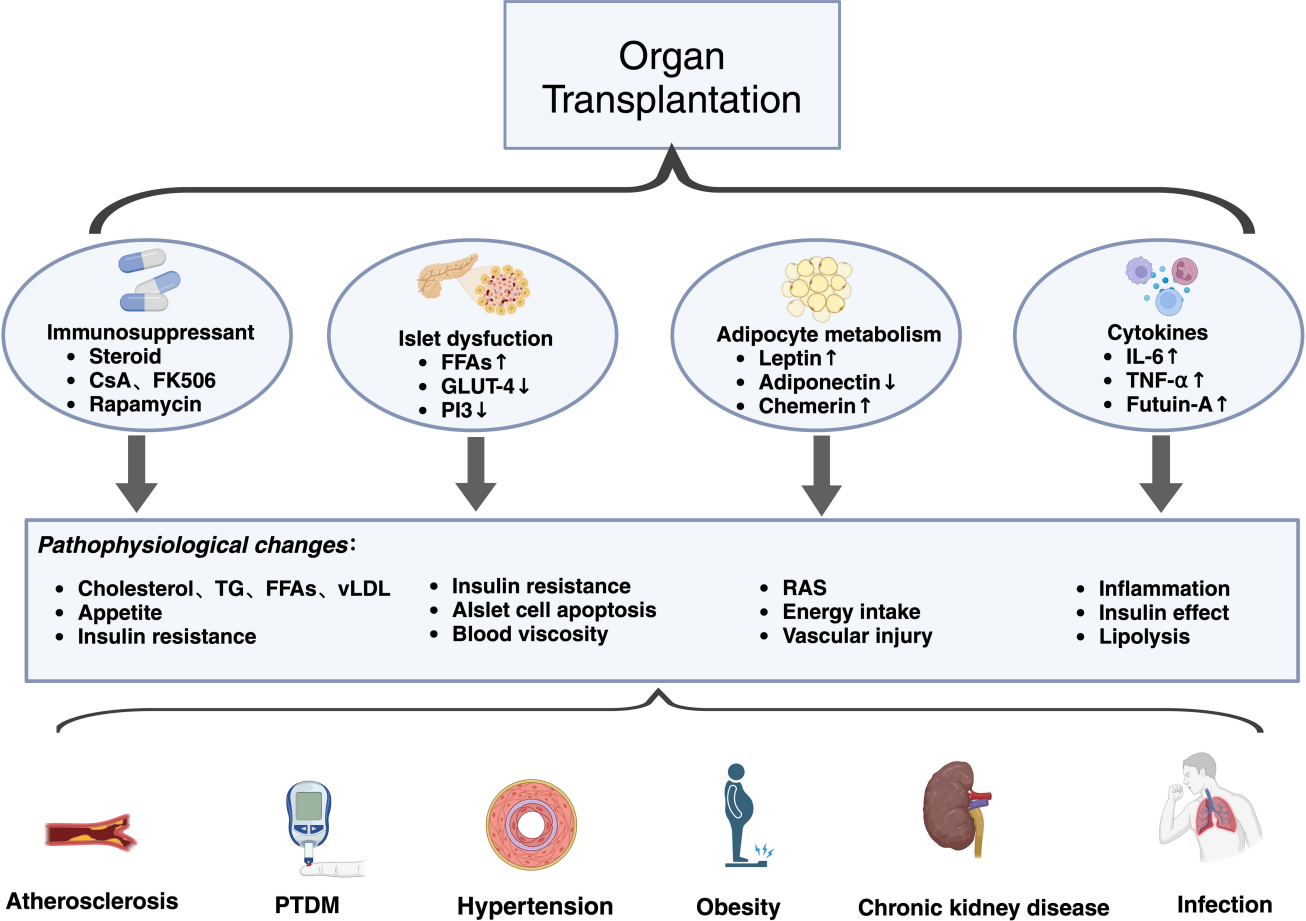
The main mechanism of MetS after transplantation. The application of immunosuppressants is one of the most important reasons for MetS. The application of steroid hormones, cyclosporine, and calcineurin inhibitors can cause abnormal lipid metabolism, increased appetite, and insulin resistance. Islets are affected by FFAs in MetS, and signals related to glucose transport are downregulated, which results in insulin production effects and hemodynamic alterations. Alterations in adipokines secreted by adipocytes further lead to vascular damage and changes in hormone sensitivity. The secretion of some cytokines can cause persistent inflammation and accelerated lipolysis. This series of changes eventually leads to adverse effects such as vasculopathy, PTDM, chronic kidney disease, and infection after transplantation.

Obesity not only causes the induction of PTDM before transplantation but is also a common complication after transplantation. Obesity can affect carbohydrate metabolism or aggravate diabetes by increasing insulin resistance after transplantation, and obesity is more likely to cause postoperative atherosclerosis (60). Increased visceral fat in obese individuals can lead to metabolic abnormalities through the secretion of inflammatory adipokines and thereby increase the risk of cardiovascular accidents (61). Weight gain before transplantation increases the rate of transplant failure, prolongs the duration of transplant surgery, and leads to proteinuria after transplantation, which eventually leads to obesity-related chronic kidney disease (**Figure 2**) (62).

## 3 Effects of MetS on the immune system

The proliferation, differentiation, and functional activity of immune cells are fundamentally regulated by the dynamic equilibrium of the immune microenvironment (63, 64). MetS perturbs this equilibrium through dysregulation of metabolites and signaling molecules via the aforementioned pathophysiological pathways. Notably, MetS significantly alerts the critical metabolic components within the immune microenvironment, including insulin signaling, carbohydrate homeostasis, lipid profiles, bile acid dynamics, and amino acid availability (1). These metabolites orchestrate immune cell differentiation and function, inducing bidirectional regulation in immune cell phenotypes toward pro-inflammatory and anti-inflammatory states (**Table 1**).

**Table 1:** Mechanism of metabolic influence on immunity.

	Effector cells	Subsets of cells	Mechanism	Related molecules and pathways	References
Abnormal Insulin metabolism	T cells	Memory T cells	Hyperinsulinism stimulates GLUT-1 expression, enhances glucose transport, and accelerates glucose uptake	GLUT-1	(73)
		CD8 T cells	Hyperinsulinemia increased the cytokine secretion of T cells under the costimulation of CD28	CD28	(75)
		Effector CD4 T cells CD8 T cells	Insulin resistance leads to the loss of INSR signaling, the downstream signaling of T cells is inhibited, and the function is down-regulated	INSR	(76)
Abnormal glucose metabolism		Memory T cells	Under hyperinsulinism, the expression of CD127 molecules, the differentiation of memory CD8 T cells and the secretion of cytokines were decreased	CD127	(73)
			Activation of the MAPK pathway enhances proliferation and effector capacity, and leads to chromatin decondensation	MAPK	(77)
	B cells	Plasma cells	Mitochondrial structure is altered, respiratory function is reduced, and plasma cell immunity is suppressed	mTOS	(81)
	Macrophages		Hyperglycemia promotes the transformation of macrophages into M1 and induces persistent inflammatory expression after epigenetic changes	MLL H3K4 methyltransferase enzymes	(82, 85)
			Epigenetic changes in macrophages induced by hyperglycemia lead to proinflammatory responses	SMYD3 SET7/9 S100A12 S100A9	(86)
			The high glucose environment induces immune memory in mitochondria	LC3bBeclin-1 RhoA/ROCK	(87, 88)
			Hyperglycemia promotes M1 transformation, inhibits M2 phenotype, and promotes inflammation development.	STAT CD163 Acsl1 Notch FoxO1 CCL18 IRF8 NF-κB/Caspase-1	(90–92, 95, 96, 98)
			Hyperglycemia induces pyroptosis of macrophages	ser-ulk1-757 mTORC1	(97)
			Under hyperglycemia, macrophage mechanosensitivity and cytokine secretion are increased	TLR4/LPS	(99)
Abnormal lipid metabolism	T cells	Treg cells	Hyperlipemia promotes the proliferation of Treg cells	FOXP3	(106, 110, 111)
			High cholesterol inhibits the proliferation and differentiation of iTreg and nTreg	HIF-1α Foxp3 mtROS	(113)
			High cholesterol inhibits Treg cells differentiation	ABCG1 p-STAT5/Foxp3	(115–117)
		Th17	High cholesterol inhibits the proliferation of Th17 cells by activating LXR	LXR ABCG1 srebp-1	(119, 120)
		CD4 T cells	High cholesterol activates LXR and enhances the T plasma membrane lipid raft profile	LXR UGCG	(118)
		Tfh cells	High cholesterol activates LXRβ, which in turn affects CD4T cells and ultimately leads to the restriction of Tfh differentiation	LXR GSK3β TCF-1	(121)
			Triglyceride stimulated Th1, Th17 and CD4 T proliferation and differentiation *in vitro*	CD36	(122, 123)
	B cells	Plasma cells	High cholesterol inhibits plasma cells differentiation through SREBP2 expression	SREBP2	(124)
			High cholesterol induces multiple pathways leading to B-cell expansion	LXRα LXRβ CD11c+ APC BM SDF-1:CXCR4 CD53/TSPAN25/MOX44	(127–129)
	NK cells		High cholesterol activates NK cells killing through multiple pathways	NCR1 NKG2D STAT ERK	(130)
			High cholesterol inhibits NK cell-related pathways	c-Myc/P300 H3K27ac	(131)
			High TG reduces CD36 expression and NK cells activity	PI3K-Akt-mTOR/FOXO1 CD36	(132)
	macrophages		Hyperlipidemia negatively regulates inflammation by inhibiting the increase of protein kinase C delta	pAkt PKCδ	(136, 137)
			Hyperlipidemia can promote the expression of CD80 on macrophages and affect NKT induced inflammation	CDd CD80	(138)
			Hyperlipidemia promotes inflammation by promoting CD36 expression and PCSK9 expression	PCSK9 CD36	(136, 139, 140)
			Hyperlipidemia induces macrophages to suppress inflammation through external vesicles	ApoE miR-146a-5 miR-142a-3p	(141)
			Hyperlipidemic endoplasmic reticulum stress promotes M1 polarization	IRE1α	(143)
			Hyperlipidemia related metabolic pathway changes	Bmp2 Cx3cl1 Tlr6	(142)
	neutrophil		Hyperlipidemia accelerates neutrophilic inflammation	BM SDF-1:CXCR4 CCR1 CCR2 CCR5 CXCR2	(128, 145, 146)
			Hyperlipidemia accelerates neutrophil mobilization	NOD1	(147)
	dendritic cells		Hyperlipidemia induces DC to express ligands to regulate the functions of NK, Treg and CD4 T cells	PD-L1 TGF-β1 NKG2D RXRA TSLP ox-LDL cDC2	(132, 148, 150, 152)
			Activation of DC by ox-LDL	TLR4/MyD88-NF-κB ox-LDL TLR IL-12p4	(149, 151)
Abnormal amino acid metabolism	T cells		Glutathione can regulate the expression of PD1 in T cells	PD-1/PD-L1	(155, 156)
			A variety of amino acids can cause T cell immune disorders	mTOR	(153, 154, 158)
		Th17 cells	Glutaminase activates Th17 to induce inflammatory response	GLS1	(160, 161)
		Memory T cells	Citrulline cycling promotes CD8 memory T cells development	Kbhb Cps1	(159)
		CD4 T cells	Homoarginine affects the protein skeleton to regulate the function of CD4T cells	Myh9	(162)
	Dendritic cells		Tryptophan regulates Treg cells by regulating DC	IDO	(157)
	macrophages		Amino acids affect mitochondria and then affect the differentiation of macrophages into M2 type		(163)
Abnormal metabolism of bile acids	T cells	Th17 cells	3-oxoLCA inhibited Th17 differentiation	RORγt	(165, 175)
		Treg cells	3-oxoLCA enhances Treg differentiation	mitoROS FoxP3	(165)
			The intestinal BA pool affects Treg cells responses	BA-VDR RORγ	(167)
			isoalloLCA enhances iTreg cells differentiation	NR4A1 mTROS Foxp3	(173)
		CD3 + CD8 + T cells	TCA inhibited CD3 + CD8 + T cells activity		(174)
		CD8 cytotoxic T cells	DCA reduced CD8 cytotoxic T cells function	PMCA NFAT2	(166)
			UDCA affects the transcription and antigen presentation of T cells	FXR TGR5	(168)
			CDCA increased the expression of FXR, and DCA inhibited the expression of FXR, which affected the activation of T cells	FXR store-operated Ca2+ NFAT	(59, 169, 170)
			CBA and Mdr1 induce immune equilibrium	Teff Mdr1	(171)
		Th1	LCA inhibits Th1	VDR ERK-1/2	(172)
	macrophages		TCA induced the transformation of macrophages into M2 type	SIRT5	(176)
			CDCA inhibited the transformation of macrophages into M2	ROS/p38 MAPK/DGAT1	(177)
			UDCA induced the transformation of macrophages into M2	NF-κB FXR	(178)
			DCA and CDCA promoted macrophage M1 transformation	NLRP3 FXR	(180)
			Multiple bile acids activate TGR5 to promote M2 transformation	TGR5	(175)
	NK cells		TCA inhibited NK cells activation	NKG2D	(174)
			TLC inhibited NK cells migration		(179)

Previous research has predominantly focused on the incidence of post-transplant metabolic syndrome (PTMS) in relation to patient survival, complications, and graft functional outcomes (65, 66). However, the impact of PTMS on immune cell dynamics and the immune microenvironment remains underexplored. Emerging evidence indicates that PTMS induces sustained immune system activation, characterized by elevated and persistent chronic inflammatory burden, which correlates with adverse clinical prognoses (67). Concurrently, PTMS-driven metabolic dysregulation promotes hyperactivation and tissue infiltration of immune cells, a phenomenon mechanistically linked to vasculopathy and hepatic complications in transplant recipients (68–71).

### 3.1 Effects of hyperglycemia and hyperinsulinemia on immunity

#### 3.1.1 Effects of hyperglycemia on T cells

Diabetes is a common complication after transplantation (72). Although hyperinsulinemia enhances the ability of memory CD8 T cells to regulate GLUT-1 and promote glucose uptake, the direct effect of hyperglycemia on memory T-cell function is predominant in diabetes. Furthermore, it can reduce the secretion of cytokines without reducing the proliferation of memory T cells or the function of memory T cells (73). Hyperinsulinemia activates effector CD8+ T cells and insulin signals through PI3K, and in this process, insulin stimulation enhances the costimulatory effect of CD28, which further enhances effector T-cell action (74, 75). In patients with insulin resistance, the downstream pathway of insulin receptor (INSR) signaling may be depleted and insulin-stimulated AKT signaling may be reduced, which may be related to a decrease in T-cell immunity (76). In hyperglycemia, increased activation of the MAPK pathway in T cells leads to enhanced cytokine production, proliferative phenotypes and chromatin decondensation (77).

#### 3.1.2 Effects of hyperglycemia on B cells

Under the influence of diabetes, the B-cell repertoire is altered, which leads to immune confusion (78). B cells present a more proinflammatory phenotype, IL-6 and TNF-a are increased, and IL-10 secretion is decreased. However, the antibody reactivity of B cells is decreased, which is not consistent with the proinflammatory phenotype of B cells (79). Another study revealed elevated levels of IgA, IgM, and total antibodies produced by B cells in the vitreous of the eye (80). Other studies have reported that insulin resistance can damage the metabolic phenotype of B cells, increase the volume of mitochondria in cells, change the structure of the cristae, and reduce the mitochondrial respiratory function of B cells. The result is that the production of plasma cells is inhibited, and B-cell immunity is reduced (81).

#### 3.1.3 Effects of hyperglycemia on macrophages

Macrophages are divided into proinflammatory M1 macrophages and anti-inflammatory M2 macrophages. Under conditions of high blood glucose, macrophages are more prone to M1 polarization. The decreased release of protective inflammatory factors, such as Arg-1 and CD206, and increased expression of proinflammatory markers, such as IL-6, iNOS, and TNF-α, as well as metabolites like the α-dicarbonyl and AGER pathways (82–84), have been reported. Hyperglycemia promotes the expression of proinflammatory M1-associated Il-6, which increases the immune capacity of macrophages. This effect is associated with metabolic changes driven by epigenetic reprogramming, which may be caused by epigenetic changes in multiple genes caused by lactic acid (82, 85). It is also possible that the expression of histone methyltransferases SMYD3 and SET7/9 upregulates the epigenetic regulation of S100A12 to upregulate the expression of S100A9 and induce an activated histone code at the corresponding gene promoters in M1 macrophages. Enhanced macrophage responses to Toll-like receptor (TLR) ligands include palmar folate (PA), lipopolysaccharide (LPS), and proinflammatory stimuli (86). In a study comparing diabetic and nondiabetic macrophages, researchers reported that the autophagy proteins, LC3b and Beclin-1, which maintain mitochondrial homeostasis in normal macrophages, were downregulated in diabetic macrophages. This phenomenon suggests that macrophages may adapt to long-term hyperglycemia and that its downregulation may reduce mitochondrial homeostasis, which is closely related to the increase in reactive oxygen species (ROS) in hyperglycemic conditions (87, 88). A study on the promotion of inflammation in macrophages by hyperglycemia revealed that macrophages take up CD163, which inhibits the uptake of Hb-Hp1-1 and Hb-Hp2-2 by macrophages and thus produces a vascular protective effect (89). Simultaneously, Arg1 and Mrc1 gene induction and subsequent STAT3 and STAT6 signaling activation are reduced (90). Another study revealed that the transcription of Acsl1 mRNA was induced in macrophages and hyperglycemia alleviated the repression of CHREBP, which further promotes Acsl1 transcription and leads to a proinflammatory response (91). Moreover, hyperglycemia can lead to the upregulation of Notch, which further promotes M1 transformation and exacerbates inflammatory injury (92). High blood glucose can induce the activation of Raw264.7 macrophages, participates in the activation of the MAPK pathway, and increases the secretion of TNF-a and macrophage adhesion (93). The activation of LPS involved in phagocytosis under high-glucose conditions led to a slight increase in the expression of the CD36 gene under normoxia and normoglycemia, and the expression of inflammatory factor-related genes was upregulated (94). Furthermore, it promoted the inflammatory phenotype of macrophages by attenuating FOXO1-activated IL-10 expression (95). M2 cells are limited in their ability to differentiate because of the inhibition of CCL18 production (96). These changes in metabolic pathways lead to the transition of macrophages to the M1 type. Researchers have shown that high glucose stimulates mTORC1 activation and ser-ulk1-757 activation, which induces pyroptosis, but these changes have no effect on macrophage activation (97). High-glucose conditions induce macrophage senescence and SASP factor secretion through NLRC4 phosphorylation, which further stimulates the NF-κB/Caspase-1 cascade through an IRF8-dependent pathway (98). Similarly, LPS enhances Acsl1 promoter activity through NF-kappa B, CHREBP and p65/RELA to promote inflammation (91). Studies have shown that the mechanical sensitivity of macrophages to high glucose is also increased. With increasing substrate stiffness, the expression of the related proinflammatory genes TLR4/LPS also increases, and the secretion of TNF-a increases (99).

### 3.2 Effects of hyperlipidemia on immunity

#### 3.2.1 Effects of hyperlipidemia on T cells

Cholesterol plays a major role in the composition and fluidity of the cell membrane and affects the differentiation and function of immune cells (100). Cholesterol metabolism is associated with T-cell proliferation (101), and sterol response element binding protein (SREBP) on the ER membrane of T cells is a sensor for T cells to sense lipids. Under low-fat conditions, it binds to SREBP cleavage activator protein (SCAP) and enters the nucleus, where it increases the expression of synthetic-related enzymes, such as hydroxymethylglutaryl (HMG)-CoA reductase (HMGCoR) and low-density lipoprotein receptor (LDLR), and also enhances endogenous cholesterol uptake. Under high-fat conditions, the insulin-inducible gene protein (INSIG) present in the ER membrane colonizes SREBP-SCAP to the ER. When the level of intracellular cholesterol is too high, liver X receptors are activated, which inhibit enzymes related to cholesterol synthesis and reduce exogenous cholesterol uptake (102, 103). After organ transplantation, normal cholesterol homeostasis is disrupted and serum cholesterol levels are elevated (104, 105). Several human and mouse studies have shown that increased serum cholesterol disrupts cholesterol metabolic homeostasis and enhances TCR activity, Treg cells development, and T-cell proliferation (106, 107). An appropriate reduction in cholesterol leads to a decrease in the proliferation and function of T cells and the secretion of IL-10 and IL-12 (108). It also changes Th1 cells to Th2/Th3 cells and increases TGF-β1 in circulation, the spleen and lesions (109). Previous studies have shown that the expression of FOXP3 in splenic Treg cells is upregulated under the influence of high cholesterol, but the proliferation of Treg cells does not inhibit the inflammation caused by T-cell activation. A likely reason is that the suppressive effect of Treg cells is limited by preactivated effector cells (106, 110, 111). However, depletion of FOXP3+ Tregs further aggravates hyperlipidemia and atherogenic inflammation (112). In contrast to previous findings, a recent study suggests that hypercholesterolemia mediates the inhibition of iTreg (inducible) cell differentiation *in vitro* by activating HIF-1α to inhibit Foxp3 and target it for proteasomal degradation. Cholesterol triggers mtROS and inhibits degradation, which strongly induces HIF-1α. This activation mode impairs the inhibitory activity of nTregs by inducing mitochondrial oxidative stress, damaging the mitochondrial structure, and inhibiting the differentiation of nTreg cells (113).

The protein levels of ABCG1 and ABCA1 are increased in cells in high-cholesterol environments, and cholesterol inhibits the degradation of these two proteins through the ubiquitin–proteasome pathway, which leads to their continuous increase in cellular content (114). These two proteins are involved in the reverse transport of intracellular cholesterol to the liver. The upregulation of the ABCG1 protein is suggested to be the cause of Treg cells suppression according to some studies. If the expression of cellular ABCG1 is too high, it leads to the downregulation of the p-STAT5/Foxp3 pathway in thymic Treg cells and the inhibition of Treg cells proliferation (115).

The functional activation of thymic TCRs and altered thymocyte differentiation, as well as Treg cells proliferation, have been observed in several studies with genotypes lacking the encoding ABCG1. This deficiency inhibits mTOR signaling and atherosclerosis (116, 117). Additionally, in the case of TCR stimulation, the loss of ABCG1 in CD4 T cells leads to excessive proliferation *in vitro*, which may cause a severe immune response (117). These results may explain why, in the context of hyperlipidemia, the activation of Treg cells was promoted but did not significantly suppress the inflammatory response.

LXR, a type α and β transcription factor, regulates T-cell proliferation and TCR activity by synthesizing oxysterols and activating the LXR transcriptional network to drive cholesterol efflux and reduce cholesterol influx and synthesis (101, 118). Current studies have shown that high cholesterol conditions increase the expression of LXR in T cells and that the activation of LXR leads to reverse cholesterol transport and inhibits the proliferation and cell cycle progression of T cells; additionally, LXR regulates T-cell activation through the ABCG1 pathway (101). In another study, overexpression of LXR α and LXR β led to a decrease in IL-17, which in turn led to a decrease in Th17. Specifically, LXR activated Srebp-1a and Srebp-1c, and srebp-1 activation affected Th17 differentiation through physical interactions with Ahr and suppressed the expression of related genes. In general, depletion of these cells effectively inhibited EAE development (119, 120). Other studies have shown that LXR activation can stimulate the transcription of the CD4+ T glycosphingolipid biosynthetic enzyme UGCG and enhance the lipid raft profile of the T plasma membrane, but additional related signaling pathways are also worth exploring (118). It has also been reported that LXR β activation inhibits GSK3β phosphorylation, downregulates TCF-1 in CD4+ T cells, inhibits Tfh cells differentiation, and attenuates the GC response and antigen-specific IgG production (121).

Triglycerides can stimulate the proliferation of CD4 T cells, the expression of IL-17 and IFN-γ, and the differentiation of Th1 and Th17 cells *in vitro* (122). Furthermore, vLDL in hyperlipidemic patients may induce atypical Th1 pathway activation through the CD36 pathway (123).

#### 3.2.2 Effects of hyperlipidemia on B cells

Previous studies have shown that diets high in cholesterol reduce B cell-mediated humoral immunity. Under high cholesterol conditions, 25-HC production is increased, and 25-HC can reduce the ability of B cells to differentiate into plasma cells by inhibiting SREBP2 gene expression and protein activation followed by entry into the Golgi apparatus to function (124). A cohort study revealed a strong inverse association between HDL cholesterol and IgD expression in B cells including naive B cells (125). Some studies have also shown that the expression of Tfr and Tfh cells is increased under a high cholesterol diet, whereas B cells are dependent on Trf to induce the expansion of Breg cells in the presence of Tfh cells (126). A high cholesterol diet leads to an increased risk of autoimmune diseases and the expansion of B cells. The loss of LXRα and LXRβ expression in CD11c+ APC could facilitate this process by increasing Baff and April in the TNF family because of a high-cholesterol diet, which leads to increased B-cell proliferation (127). Additionally, high cholesterol disrupts the SDF-1: CXCR4 axis in BM and promotes B-cell mobilization (128). Under high-fat loading conditions, high-fat exposure and inflammatory stimuli induce CD53 *in vivo* in the liver and isolated primary hepatocytes. The transmembrane tetraspanins CD53/TSPAN25/MOX44 mediate B-cell development and lymphocyte migration to lymph nodes (129).

#### 3.2.3 Effects of hyperlipidemia on NK cells

In mice fed a high-cholesterol diet, the number of NK cells is increased, and the expression of molecules related to NK cells activation receptors, cytokines and effector molecules that play cytotoxic roles is increased (130). Another study reported that lipid accumulation inhibited the killing of NK cells by reducing c-Myc/P300 and H3K27ac (131). In hyperlipidemic conditions, especially hypertriglyceridemia, the expression of CD36 on NK cells is increased and the PI3K-Akt-mTOR/FOXO1 signal transduction axis is downregulated. Low mTOR activity represents low glycolysis/FAS and reduced cell growth and function. Moreover, lipids express PD-L1, TGF-β1, and NKG2D ligands to reduce NK activity (132).

#### 3.2.4 Effects of hyperlipidemia on macrophages

Macrophages are important cells in hyperlipidemia and affect atherosclerosis. They are also sensitive to different lipids and differentially release inflammatory factors (133). Hyperlipidemia can lead to an increase in the M1 proinflammatory phenotype and a decrease in the M2 anti-inflammatory phenotype (134). Recent studies have further addressed the effects of hyperlipidemia on macrophages. One study suggested that excess intracellular cholesterol decreased ABCA1 expression and the upregulation of ABCA5 was the main cause of cholesterol efflux (135). Another study reported that hyperlipidemia can promote the restriction of PKCδ isoform activation in macrophages, which reduces its number and inflammatory response in the arterial wall (136). Among the coactivated isoforms, PKCδ activation has a proapoptotic effect on monocytes/macrophages (137). Hyperlipidemia promotes the activation of CD80+ macrophages, which further promotes the development of NKT cells by increasing CDd expression and accelerating early atherosclerosis (138). CD36 expression is increased in hyperlipidemic patients and can affect lipid metabolism in macrophages (136). One possible mechanism is that PCSK9 expression and CD36 expression increase inflammation (139), but further studies are needed to investigate the role and mechanism (140). Under hyperlipidemic conditions, the expression of macrophage-activating ApoE can be initiated by miR-146-a-5p vesicles, and miR-142-a-3p controls the transcription axis and thereby inhibits inflammation (141). Recent studies on macrophage immunity in hyperlipidemic heart failure have revealed that genes associated with lipid transport are downregulated and glycolysis and fatty acid synthesis are upregulated in myocardial resident macrophages in hyperlipidemic heart failure models. Bmp2, Cx3cl1 and Tlr6 were also found to be upregulated, which is consistent with the activation of the NF-kB signaling cascade according to GSEA. In contrast to previous studies, Car3 was significantly downregulated in the cardiac macrophages of HFD-fed mice (142). Under high-lipid conditions, ER stress induces IRE1α activation, inhibits M2 polarization and enhances M1 polarization (143). Furthermore, lipid overload is mediated through the endoplasmic reticulum stress pathway. Studies on the macrophage–cardiomyocyte regulatory axis reveal the potential effects of a variety of inflammatory cytokines secreted by macrophages on cardiomyocytes, especially on pathways such as hypertrophy, fibrosis, and autophagy (144).

#### 3.2.5 Effect of hyperlipidemia on neutrophils

Hypercholesterolemia can induce an increase in neutrophils, which is possibly due to stimulation of granulopoiesis, enhanced bone marrow mobilization, and reduced peripheral clearance. Increased numbers of neutrophils adhere to arteries via the chemokines CCR1, CCR2, CCR5 and CXCR2 and cause early atherosclerotic lesions (145). CXCR2 in hyperlipidemic patients can aggravate brain damage caused by neutrophils after cerebral ischemia (146). One study showed that high-fat conditioning can stimulate splenic NOD1 and activate neutrophil recruitment, activation and function (147). Other studies have shown that high cholesterol disrupts the SDF-1:CXCR4 axis in the BM and accelerates neutrophil mobilization (128).

#### 3.2.6 Effects of hyperlipidemia on dendritic cells

Hyperlipidemia can induce the expression of PD-L1, TGF-β1 and NKG2D ligands in lipid-loaded DCs, which is related to NK cell activation (132). In addition, hyperlipidemia can increase ox-LDL and then activate retinoid X receptor α (RXRα) and IL-1β to attenuate TSLP expression in cultured thymic epithelial cells (TECs). This activation results in the suppression of LAP and PD-L1 expression in DCs, which leads to defective Treg function (148). Under hyperlipidemic conditions, an increase in ox-LDL can lead to the activation of the TLR4/MyD88-NF-κB signaling pathway in DCs and enhance their migration, maturation and secretion of inflammatory cytokines (149). Hypercholesterolemia causes rapid changes in DCS in the bone marrow, and unlike PDCS, the spleen and lymph node cDC2 subsets accumulate lipids. Although lipid-loaded cDC2s increase costimulatory molecule expression and cytokine production, their ability to initiate naive CD4 T cells is significantly impaired (150). Dyslipidemia inhibits Toll-like receptor (TLR)-induced DC activation and related inflammatory cytokine production, mainly because oxLDL upregulation inhibits TLR-induced IL-12p40 production in CD8α− DC subsets. Inhibition of this subset results in impaired Th1 and enhanced Th2 responses (151). It has also been suggested that the enhancement of CD11c+ dendritic cells is beneficial to the function of CD4+ T cells under hyperlipidemic conditions (152).

### 3.3 Effects of amino acid metabolism on immunity

Amino acid metabolism can regulate T-cell function by regulating the mTOR signaling pathway. A variety of amino acids can regulate mTOR activity and selectively activate mTORC1 and mTORC2 to regulate T-cell differentiation. However, the general control nonsuppressor 2 (GCN2) signaling pathway regulates T-cell differentiation in a manner independent of amino acid activation (153, 154). The same amino acids can also affect the immune response through the PD-1/PD-L1 pathway. Glutathione can activate PD-L1 in tumor cells to inhibit T-cell activity, and the loss of PD-1 can induce immune tolerance (155, 156). In tryptophan metabolism, dendritic cells expressing IDO can increase the function of Treg cells through the tryptophan metabolism pathway (157). Amino acid metabolism disorders are related to the occurrence of elevated uric acid. Studies have shown that exposure to bacterial amino acid metabolism disorders can lead to elevated uric acid, which in turn leads to gout. This metabolic disorder leads to immune abnormalities in intestinal T cells through the mTOR signaling pathway (158). Moreover, CD8-positive memory T cells can clear ammonia and promote memory cell development through urea and citrulline cycles (159). Studies on cancer and psoriasis have revealed that the activation of GLS1, which is the GLS isoform, enhances the Il17a promoter, promotes the proliferation of Th17 cells, and leads to immune inflammation and psoriasis. GLS loss is the cause of tumor immunosuppression (160, 161). Homoarginine strongly regulates the actin cytoskeleton of T cells by inhibiting myosin heavy chain 9 (Myh9) and thereby affects the function of CD4 T cells. Homoarginine also inhibits the proliferation of T cells and the migration of related chemokines (162). In macrophages, the metabolism of some amino acids also leads to the differentiation of M2 macrophages through the influence of mitochondria (163).

### 3.4 Effects of bile acids on immunity

#### 3.4.1 Effects of bile acid metabolism on T cells

Bile acids play an important role in metabolic homeostasis. The Fxr, Shp, Lxr and Srebp1c genes, which are related to bile acid metabolism, are closely related to inflammation and immune hyperexcitability (164). 3-oxoLCA inhibits Th17 cell differentiation by direct physical binding to its key transcription factor, retinol-related orphan receptor γt (RORγt), while isoalloLCA enhances Treg differentiation by producing mitochondrial reactive oxygen species (mitoROS), which leads to increased FoxP3 expression. Isoallolca-mediated Treg enhancement requires an intron-based FoxP3 enhancer, the conserved noncoding sequence 3 (CNS3), which is a distinct mode of action compared to previously identified Treg-enhancing metabolites that require CNS1 (165). Microbial metabolites in the gut can affect the systemic immune environment. Primary bile acids in the gut are metabolized by microorganisms into secondary bile acids. Deoxycholic acid (DCA) is the most abundant secondary bile acid in the human body and largely reduces the proportion of CD8 cytotoxic T cells and IFN-γ-producing cells. The mechanism involves DCA enhancing PMCA-mediated Ca efflux and reduces the intracellular Ca concentration, whereas low intracellular Ca inhibits NFAT2 signal transduction and inhibits CD8 T cell effector function (166). Dietary and microbial factors can affect the composition of the intestinal BA pool, affect the BA-VDR axis and regulate the expression of the transcription factor RORγ in colonic FOXP3 regulatory T cells (Tregs). RORγ+ Tregs play a role in anti-inflammatory effects and inhibit excessive immune responses during the inflammatory response (167). GVHD also alters the BA pool and shows a decrease in most bile acids and an increase in ursodeoxycholic acid (UDCA) and intestinal Nr1h4 mRNA (encoding FXR) but a decrease in Gpbar1 (encoding TGR5). These changes affect the transcription and antigen presentation capacity of intestinal T cells. However, the effect on systemic T-cell expansion is not clear (168). T cell-induced inflammation reduces the abundance of microbial-derived bile saline hydrolase (BSH) genes and increases the expression of FXR. This effect on the BA pool leads to an increase in bile acids, such as chenodeoxycholic acid (CDCA), which promote FXR receptor activation and a decrease in DCA that inhibits FXR activation. In this study, ursodeoxycholic acid (UDCA) was found to reduce FXR activation, effector T-cell activation and graft-versus-host disease (59, 169). UDCA and DCA disrupt the intracellular Ca concentration through mechanisms that inhibit Ca uptake by mitochondria and increase cytosolic Ca, which leads to STIM1 and ORAI1 uncoupling and impairing store-operated Ca+ entry. This process is essential for NFAT signaling and T-cell activation, which in turn decreases T-cell activation (170). Studies have shown that the loss of MDR1 (ABCB1) function in the inflammatory response indirectly leads to the upregulation of Teff cells, which secrete cytokines and aggravate the inflammatory response. Mdr1 functions in the presence of conjugated bile acid (CBA), which alleviates oxidative stress and maintains immune balance in Teff cells (171). Unconjugated glycocholic acid (LCA) can block the activation of Th1 cells. The mechanism may involve the activation of the vitamin D receptor (VDR) after increasing the LCA concentration, after which the phosphorylation of ERK-1/2 is inhibited to reduce the activation of Th1 cells (172). IsoalloLCA is an isoform of LCA that is transformed in intestinal bacteria. It can induce the mitochondrial production of mtROS and then induce NR4A1 recruitment to the Foxp3 gene promoter to stimulate Foxp3 gene expression and enhance iTreg cell differentiation (173). TCA can inhibit the activity of CD3+CD8+ T cells and reduce their number both *in vitro* and *in vivo*, but the specific mechanism still needs to be explored (174). While 3-oxoLCA and isoLCA inhibited Th17 cell differentiation *in vitro*, DCA and LCA inhibited Th17 cell differentiation only *in vivo* (175).

#### 3.4.2 Effects of bile acid metabolism on macrophages

SIRT5 is an important metabolic regulator. Recent studies have shown that the absence of SIRT5 leads to an increase in bile acids, mainly taurocholate (TCA), in hepatocytes and increased TCA leads to the M2-type transformation of macrophages (176). Chenodeoxycholic acid (CDCA) was determined to inhibit macrophage M2 transformation in an allograft model (177). In another study, the anti-inflammatory effect of ursodeoxycholic acid (UDCA) was partially mediated through FXR activation and NF-κB inhibition, which led to macrophage transformation from M1 to M2 (178). During the process of macrophage activation, taurine cholate (TLC) downregulates several genes related to early macrophage activation and upregulates several genes that suppress immunity and stabilize macrophages. These findings indicate that TLC has multiple effects on macrophages and its effect on macrophages needs further study (179). DCA and CDCA can significantly increase the level of NLRP3 in macrophages. This activation is dependent on calcium influx into the mitochondria, which in turn promotes the transformation of macrophages to an inflammatory type. However, overexpression of FXR inhibited NLRP3 expression, which contradicts the activation of NLRP3 by FXR ligands (180). Moreover, previous studies have suggested that the activation of FXR can aggravate the immune response (59), which suggests that the role of FXR needs to be further studied. It has also been reported that LCA, DCA, 3-oxoLCA and isoLCA can activate TGR5 and promote the transformation of M2 macrophages, which inhibits the activation of Th17 cells and the inflammatory response (175).

#### 3.4.3 Effects of bile acid metabolism on NK cells

Taurocholic acid (TCA) reduces the activity and number of NK cells *in vivo* and *in vitro* and thereby inhibits their response to TNF-a; however, the specific mechanism, which may be related to NKG2D expression, still deserves further study (174). The presence of TLC has been found to inhibit the gene expression of chemokines involved in NK cell migration, which also requires macrophage activation (179).

## 4 Effects of MetS on organ transplant rejection

**Figure 3:**
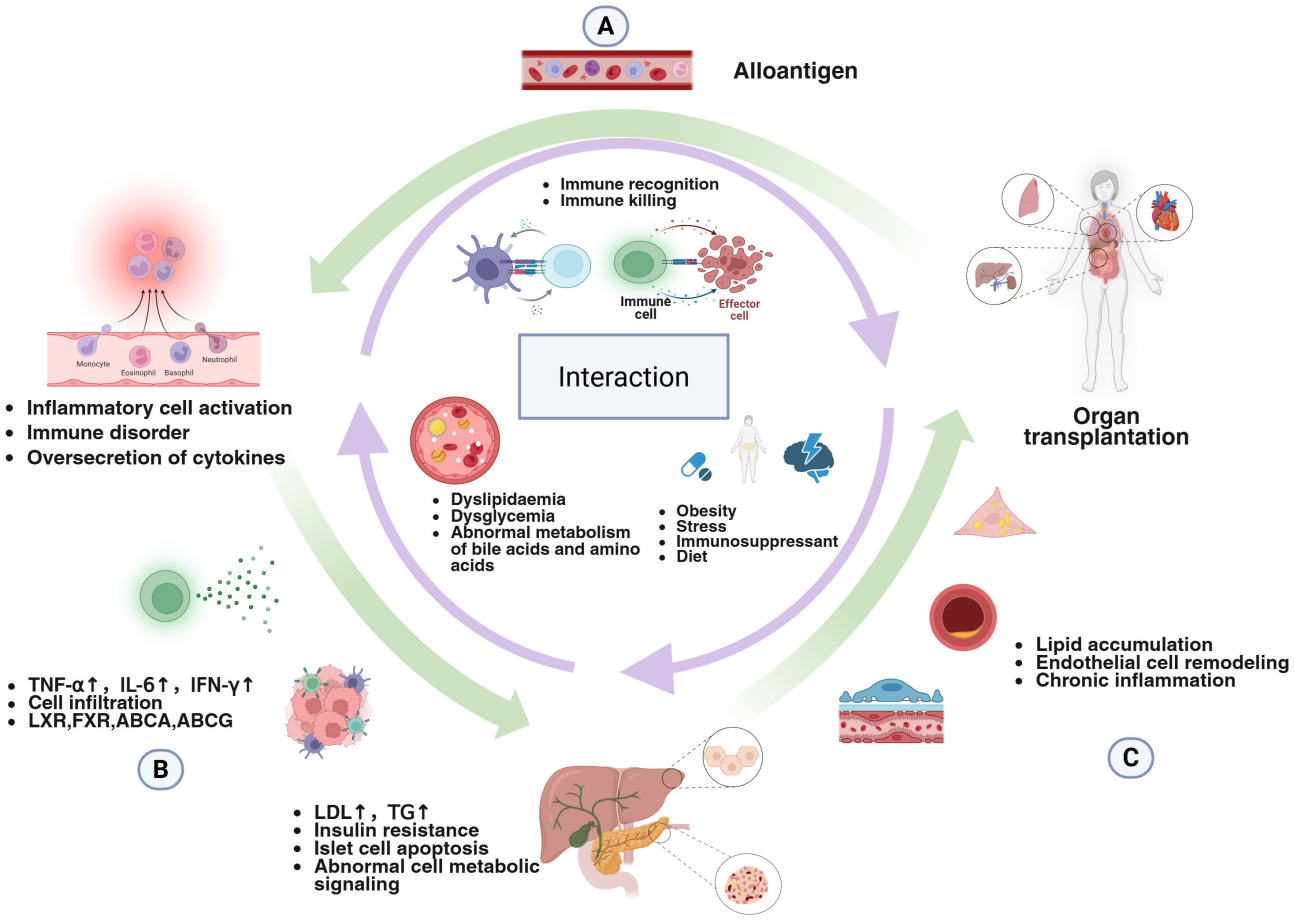
Transplantation, metabolism and immunity interact in different ways in the body. **(A)** The presence of the graft introduces alloantigens to the recipient immune system, which allows the immune system to specifically recognize and kill the graft cells. Suppression of the host's immune destruction of the graft is the most important problem in the late stage of transplantation. **(B)** The activated immune system increases the release of the cytokines TNF alpha, IL-6, and IFN-gamma; these cytokines cause immune system disorders and increase the number of inflammatory cells. This inflammation affects the molecular pathways related to metabolism and promotes the occurrence of metabolic disorders. These metabolic abnormalities further induce inflammatory changes in the immune system, disrupt the immune balance and affect the basic functions of the immune system. **(C)** Stress, immunosuppressive drugs and diet after transplantation directly or indirectly lead to the abnormal metabolism of lipids, sugars, amino acids and bile acids. This metabolic abnormality in turn affects the survival of transplant patients through cellular lipid accumulation, chronic inflammation, and endothelial remodeling.

After transplantation and due to a high-energy diet, that supplies needed macronutrients for recovery, side effects of immunosuppressive drugs, and reasons for reduced consumption, the incidence of obesity, nonalcoholic liver cirrhosis and metabolic disorders is significantly greater than that in the general population. Hyperlipemia, diabetes mellitus, and abnormal amino acid metabolism occur in most patients, and MetS is considered an important factor affecting the prognosis of transplant patients (53, 181, 182). Previous studies have demonstrated that patients developing MetS following cardiac, hepatic, or renal transplantation exhibit elevated inflammatory markers compared to non-MetS recipients. This systemic chronic inflammation, intrinsically linked to allograft rejection, mediates endothelial injury and exacerbates oxidative stress. Consequently, these pathophysiological alterations result in an elevated risk of cardiovascular events and accelerated allograft injury (**Figure 3**) (183–186).

### 4.1 Lipid metabolism and effects of transplantation

The most important effect of lipid metabolism after transplantation is an increase in cardiovascular disease (CVD) risk, and hyperlipidemia after transplantation is more likely to lead to coronary atherosclerosis (54). This particular vasculopathy is characterized by progressive narrowing of the graft vessel, which occurs after endothelial transplantation-related injury. Owing to the chronic immune response, the activation and interaction of related immune cell cascades leads to endothelial cell remodeling. Hyperlipidemia can also aggravate vascular stenosis by affecting endothelial cell function through a reduction in nitric oxide (NO) availability (187). The high incidence and serious consequences of CVD after liver transplantation make it a high concern among postoperative complications. Hyperlipidemia after a liver transplant is caused mainly by immunosuppressants (188). The relationship between hyperlipidemia and atherosclerosis has been established by many studies (136, 145), and the current consensus is to protect the cardiovascular system and reduce CVD by lowering lipids (189). In a study of heart transplantation, the levels of triglycerides and ceramides in heart tissue (at the level of cardiomyocytes) increased after transplantation, and lipid accumulation in cardiomyocytes led to a reduction in both systolic and diastolic heart function (190).

Current studies on effects of hyperlipidemia on immune mechanisms have shown that high cholesterol promotes the proliferation of effector immune cells and enhances inflammation and rejection after transplantation (100, 106, 107, 128). Additionally, the pathways by which helper T cells and Treg cells control inflammation and proliferation are inhibited (113, 115, 117). This effect is achieved mainly through the activation or inhibition of LXR, ABCG, CD36, PCSK9 and other pathways under hyperlipidemic conditions (101, 115, 119, 123, 139, 140). Upon rejection, the induced lipid antigen-presenting CD1 protein and specific T cells that are predicted to recognize CD1b and CD1c proliferate vigorously and infiltrate the graft (191). Hyperlipidemia exacerbates allograft rejection by promoting macrophage polarization toward the pro-inflammatory M1 phenotype while suppressing M2 differentiation, mediated through endoplasmic reticulum stress and downregulation of ABCA receptor expression (135, 139, 143). Concurrently, upregulated CD36 expression directly enhances macrophage cytotoxic activity, further amplifying rejection (139). Conversely, dendritic cell-mediated immune rejection is potentiated by elevated ox-LDL levels and increased CD11c+ dendritic cell populations, which act via both direct and indirect mechanisms (148, 152). This dendritic cell activation cascade drives B cell proliferation, thereby intensifying immune-mediated graft injury (127). This series of studies revealed that the influence of lipids on immune rejection is significant and that their related targets constitute future research directions for novel immunosuppressants.

### 4.2 Effects of glucose metabolism on transplantation

The incidence of diabetes after liver transplantation can be as high as 30%. Early hyperglycemia can be caused by stress due to surgery, postoperative infection, or steroid hormone use. Abnormal glucose metabolism for more than 45 days is considered new-onset diabetes, which is related to the use of immunosuppressive agents and is also known to lead to CVD (188, 192). A clinical study revealed that posttransplant diabetes mellitus (PTDM) does not affect graft survival, whereas PTDM damages the kidney, reduces the renal function of the transplant recipient, increases blood lipids, and affects blood pressure, which affects the long-term survival of patients (55). Post-transplant diabetes abnormalities are also associated with CVD, and the presence of PTDM increases the relative risk of death from CVD in transplant patients by 1.5-3 times (193). Systemic insulin resistance, which is caused by immunosuppressive agents, occurs after transplantation (2). PTDM can lead to lipid accumulation in cardiomyocytes and then to the development of cardiomyopathy before coronary heart disease; however, this abnormal metabolism can be alleviated by metformin treatment (190). In addition, an increase in the level of macrophage-activated soluble CD163 (sCD163) was detected after transplantation. An increase in this marker implies disordered carbohydrate and lipid metabolism and increased inflammation, which is associated with the progression of MetS after transplantation and affects the survival of transplant patients (194). The occurrence of PTDM increases the infection rate of hospitalized patients, especially the CMV infection rate, which is significantly higher than that of transplant patients with stable blood glucose. Diabetes is a strong predictor of donor cardiac rejection and death, but other diabetes-related complications, such as microangiopathy after PTDM, are comparable to those of nontransplant diabetes (195). Current studies on diabetes and transplantation have shown that hyperglycemia not only promotes or inhibits immune cells in general but also has different effects on immune cells under different conditions. For example, hyperglycemia reduces the function of memory T cells under certain conditions (73), downregulates T-cell immunity (76), and both promotes and inhibits B cells (79, 81). Hyperglycemia exacerbates transplant rejection by augmenting cytokine production in T cells through upregulation of the MAPK signaling pathway (77). However, studies on macrophages have generally shown that hyperglycemia promotes macrophage transformation to the M1 phenotype, which is a proinflammatory response (82–84). Research pathways have focused mainly on PI3K/AKT, CD28, MAPK, STAT, NF-κB and epigenetics. Mechanistically, hyperglycemia drives macrophage phenotypic switching via coordinated upregulation of Notch, SMYD3, MAPK, and IRF8 pathways coupled with downregulation of Arg1 and Mrc1 pathways. This transcriptional reprogramming enhances macrophage activation within the allograft microenvironment, potentiating their pro-inflammatory and cytotoxic functions to aggravate immune-mediated graft injury (74, 75, 82, 85, 91, 93, 98). Contact-related research on blood sugar and transplant rejection is also limited, and research on the immunosuppressive mechanism is not clear.

### 4.3 Effects of amino acid metabolism on transplantation

In a renal transplant study, the concentrations of BCAA (leucine, isoleucine, and valine) in patients who developed PTDM were consistently greater than those in posttransplant non-PTDM subjects, and the concentrations of branch-chain amino acids and tyrosine in posttransplant subjects were greater than those in pretransplant subjects. The elevation of these amino acids was highly correlated with the development of posttransplant diabetes. This finding also indicates the possibility of insulin resistance but the related mechanism needs further study (196). Other studies have shown that the low amino acid content after transplantation is the cause of transplantation stress. The main reason is that the activation of ERK and AKT promotes the activation of mTORC1 and the consumption of amino acids in serum, whereas the inhibition of this pathway stabilizes stress after transplantation (197). Pretransplant sarcopenia predicts a decrease in amino acid levels after transplantation, which leads to a poor outcome after transplantation and suggests that protein and amino acid supplementation after transplantation is an important nutritional requirement (198–200).

The direct effect of amino acids on the immune system through the PD-1/PD-L1 pathway is also a major research direction of transplant rejection. Determining whether deficiency or an increase in related amino acids has a protective or destructive effect on transplant patients needs to be further studied. However, according to current studies, a stable serum amino acid balance still has a protective effect on transplantation (155–157, 163, 197).

### 4.4 Effects of bile acid metabolism on transplantation

Disorders in bile acid levels after transplantation are another important factor affecting survival time after organ transplantation. The inflammatory response after transplantation disrupts the balance of bile acids, increases the content of bile acids that promote immune rejection, and exacerbates the rejection of transplantation (59, 169). Prophylactic bile acid therapy was found to reduce the severity of acute graft-versus-host disease and inhibit intestinal cell apoptosis (168). In nonalcoholic steatohepatitis studies, dysregulation of bile acids was found to lead to hepatocyte apoptosis via DR5. Deoxyursodeoxycholic acid (NorUDCA) stabilizes this metabolic disorder and improves symptoms by downregulating the FXR receptor (201). The content of fecal bile acids after transplantation is related to the prognosis of patients, especially the risk of future infection. The type and content of bile acids can determine the mucosal immune capacity and resistance of pathogens to antibiotics (202).

In bile acid metabolism, the presence of secondary bile acids, such as 3-oxoLCA, DCA, and CDCA, reduces inflammation and rejection (165, 166). However, it is not known whether the resulting immunosuppression is specific and whether such use may aggravate posttransplant infections or further aggravate disorders of carbohydrate and lipid metabolism. However, the role of the related receptor FXR and its ligands in immunity is still controversial, which also makes bile acid metabolism a hot topic in the research of posttransplantation metabolism.

Current studies have shown that the main response caused by bile acid pool disorders in MetS patients after transplantation is to promote immunity, and the treatment of bile acid disorders improves GVHD (59, 168, 169); however, studies in which ursodeoxycholic acid and NorUDCA are used to improve this disorder of bile acid metabolism still have limitations (59, 201). Disruption of the BA pool reduces BSH abundance and upregulates FXR expression, thereby promoting T cell expansion and driving a pro-rejection phenotypic shift (59, 169). Concurrently, loss of MDR1 function amplifies oxidative stress in effector T cells, exacerbating immune-mediated rejection (171). Furthermore, elevated levels of CDCA and DCA, coupled with reduced UDCA, suppress M2 macrophage polarization while promoting M1 polarization. This phenotypic reprogramming enhances macrophage-mediated pro-inflammatory activity, thereby aggravating allograft injury (178). In the future, more research is needed to stabilize bile acid disorders and reduce transplant rejection.

## 5 Management of MetS after transplantation

### 5.1 The impact of immunosuppressants on MetS

#### 5.1.1 corticosteroids

Glucocorticoids exert the most profound metabolic impact in post-transplant patients, primarily through direct impairment of postprandial tissue glucose uptake, promotion of lipolysis, and elevation of serum triglycerides and free fatty acids. These mechanisms collectively exacerbate insulin resistance, leading to glucose intolerance and dyslipidemia (203). Furthermore, chronic glucocorticoid administration induces hypertension via fluid retention and endothelial dysfunction. Notably, while discontinuation mitigates metabolic disturbances, it concomitantly elevates rejection risk due to rebound immune activation (204).

#### 5.1.2 Mammalian target of rapamycin inhibitors

Mammalian target of rapamycin inhibitors (mTOR inhibitors) predominantly disrupt lipid metabolism in post-transplant patients by elevating circulating triglycerides and free fatty acids. This metabolic perturbation is mediated through impaired APOB100 degradation, leading to elevated triglyceride accumulation (205). Furthermore, mTOR inhibitor administration contributes to insulin resistance and diabetes pathogenesis, primarily mediated through dual mechanisms: interference with insulin signaling transduction pathways such as the IRS-1/PI3K/Akt cascade and suppression of pancreatic β-cell insulin secretory capacity (206–208).

#### 5.1.3 Calcineurin inhibitors

Calcineurin inhibitors (CNIs), such as cyclosporine, predominantly disrupt lipid metabolism by inducing hyperlipidemia, marked by significant elevation of total cholesterol, low-density lipoprotein cholesterol (LDL-C), and triglycerides (209). In contrast, tacrolimus exhibits a higher diabetogenic propensity, primarily attributable to elevated FK506-binding protein 12 (FKBP12) levels in pancreatic β-cells, which directly suppresses insulin secretion (210, 211).

#### 5.1.4 Strategies for immunosuppressants adjustment

In the context of glucose dysregulation induced by immunosuppressive agents, glucocorticoid therapy is recognized to promote hyperglycemia; however, steroid withdrawal has not been shown to reduce the risk of PTDM (212). Studies indicate that maintenance therapy combining CNIs with glucocorticoids achieves an optimal balance between PTDM risk and acute rejection prevention. Notably, switching from tacrolimus to cyclosporine within CNI regimens significantly improves glycemic control without increasing acute rejection rates (213). Furthermore, concomitant use of mTOR inhibitors with CNIs does not elevate PTDM risk. mTOR inhibitor co-administration allows CNI dose reduction, thereby ameliorating glucose metabolism disturbances (214).

In transplant recipients exhibiting persistent hypercholesterolemia under CNI therapy, studies demonstrate that substituting cyclosporine with tacrolimus represents an effective therapeutic strategy (215). Hyperlipidemia induced by mTOR inhibitor administration is highly prevalent. If lipid levels remain suboptimal despite lipid-lowering agents, dose reduction of mTOR inhibitors and/or substitution with CNIs or alternative immunosuppressants is clinically warranted (188).

### 5.2 Diet and medication management

The evaluation of different risk factors in patients with MetS should achieve different goals with different strategies (216). Presently, the treatment of MetS is based on two main approaches as follows: drug management and diet management. Traditional dietary management models mainly include Med DIET, DASH, vegetarian, low-fat, low-carbohydrate and other models, and exercise can reduce the incidence of MetS after transplantation (**Table 2**). This comprehensive management helps maintain metabolic stability in patients with MetS (213, 217). The second type of drug management involves the use of statins, insulin, and oral antidiabetic drugs to control MetS (**Table 3**) (218).

**Table 2:** Dietary management of MetS.

Dietary patterns	Specific measures	Expected Results	References
Mediterranean Diet	• 35%-45% from fat, mainly unsaturated fatty acids; 35%-45% water and 15%-18% protein	• Reduce the incidence of cardiovascular disease and T2DM; reduce the blood pressure	(217, 222)
The DASH Diet	• 27% from fat, 55% from carbohydrate, 18% from protein, and some dietary cholesterol intake	• Reduce blood pressure, reduce the incidence of T2DM, and protect the heart	
Ketogenic Diet	• 20% to 30% from protein, <50% carbohydrate intake, and 30% to 70% fatty acid intake	• Decrease LDL and triglyceride; Increase HDL	
Plant-based Diet	• Eat plant-based foods and reduce animal-based foods	• Reduce blood pressure; reduce the incidence of coronary heart disease	

**Table 3:** Pharmacologic management of MetS.

Type	Management style	Drugs	Advantages	Disadvantages	References
Blood glucose management after transplantation	**Recommended level of control:Hba1c <7% (53 mmol/mol); no retinopathy or proteinuria;**				
	Hypoglycemic Agents	Insulin	• Early control • Intravenous injection followed by subcutaneous injection		(185, 220, 223, 224)
		GLP-1	• More suitable for patients with hyperglycemia and obesity • helps to reduce body weight		
		Metformin	• Adverse effects were mild	• Caution in patients with renal failure	
		Sulfonylureas	• High safety	• drug interactions; • Cause low blood sugar	
		Glinides	• High safety		
		SGLT-2 inhibitors		• Causes hypovolemia • Increases the risk of genitourinary infections	
		Thiazolidinediones	• High safety		
	Replacement of immunosuppressive drugs	Cyclosporine replace Tacrolimus	• Reduce the incidence of PTDM	• Not as first-line treatment • It may affect immunosuppression and should only be considered in severe cases	
		mTOR inhibitor replace Tacrolimus			
Lipid management after transplantation	**Recommended level of control: LDL-C<100 mg/dL and triglyceride concentration <250 mg/dL**				
	Lipid-lowering Agents	Statins	• Well tolerated and effective	• Muscle toxicity and liver toxicity	(185, 219, 225, 226)
		PCSK9 inhibitors	• Excellent lipid lowering effect	• Not as a first choice	
		Fish oil (omega-3 fatty acids)	• Safety and minimal drug interactions		
		Fibrates	• Elevate CNI levels • Use when triglycerides > 1000 mg/dL	• Nephrotoxicity	
		Ezetimibe	• High safety	• Weak lipid-lowering effect	
		Fibrous acid derivative	• Good tolerance	• Risk of rhabdomyolysis	
	Replacement of immunosuppressive drugs	Reduce Calcineurin inhibitors (CNI)	• Reduce the degree of lipid metabolism disorder caused by drugs		
		Reduce mTOR Inhibitors			
		Change cyclosporine into tacrolimus			
Blood pressure management after transplantation	**Recommended level of control: Blood pressure < 130/80 mmHg**				
	Blood Pressure Medications	Calcium channel blockers	• First-line drugs		(188)
		ACEI\ARB	• First-line drugs • Reduce proteinuria and cardiac afterload	• Exacerbating hyperkalemia	
		Beta-blockers	• Second-line Drugs		
		Loop diuretics	• Second-line Drugs		

#### 5.2.1 Glycemic management

PTDM requires individualized therapy with a combination of diet, insulin, and oral hypoglycemic agents. Metformin stands out as an oral drug because it does not bind to proteins and remains stable in terms of excretion. When GLP-1 receptor agonists are used, their interaction with immunosuppressive agents should be considered. Studies have shown that the use of vildagliptin is safer after transplantation and that GLP-1 inhibitors are more suitable for obese patients. Insulin is more suitable for oral drugs when blood glucose cannot be controlled (53, 185, 213). The replacement of immunosuppressive agents is not the first choice for PTDM. Current studies have shown that the effect of replacing immunosuppressive agents is limited, and more long-term and comprehensive studies are needed. This therapy should be considered only for severe drug-induced PTDM reactions (213).

#### 5.2.2 Management of hyperlipidemia

Statins have been shown to be effective and well tolerated, fibrates are effective but partially nephrotoxic, and ezetimibe has been shown to be safe in liver transplant recipients (185). However, statins are metabolized mostly by cytochrome P450-3A4 and so calcineurin inhibitors may be reduced. For persistent hyperlipidemia, a change in the use of immunosuppressive agents, such as cyclosporine, to tacrolimus or a reduction in the dose of mTOR should be considered (188, 219). Fish oil omega-3 fatty acids are partially effective in lowering blood lipids in isolated hypertriglyceridemia, and cholesterol levels should be monitored continuously during treatment with fish oil (185, 188).

#### 5.2.3 Weight management

Weight management advocates a combination of diet and exercise rather than the use of drugs because weight-loss drugs can antagonize immunosuppressive agents in certain pathways. Bariatric surgery should be considered in special cases when it is difficult to reduce weight with diet and exercise. Overall, weight loss significantly reduces the mortality of transplant patients (53, 220).

### 5.3 Significance of MetS management

The benefits of effective management of MetS are unequivocal in both mitigating immune-mediated rejection and sustaining metabolic homeostasis. Maintaining circulating glucose, lipids, and amino acids within optimal ranges in transplant recipients reduces the proliferative and differentiation capacity of immune cells, stabilizes the immune microenvironment, and attenuates chronic inflammatory responses. Furthermore, these sustained beneficial effects may reduce the immunosuppressive burden and improve long-term quality of life by preventing metabolic toxicity-induced complications.

## 6 Conclusions and perspectives

The mechanisms underlying the relationship between metabolic syndrome (MetS) after transplantation and immune tolerance remain unclear, and its impact on immune system function has yet to be definitively confirmed by many studies. Current immunotherapy regimens have notable limitations. Low doses are often ineffective, while high doses may lead to complications such as tumors, infections, and MetS. As a result, the search continues for treatments that not only promote metabolic stability and reduce complications but also induce immune tolerance and improve long-term quality of life for transplant recipients (221). One promising approach is the use of the new generation of lipid-lowering drugs, such as PCSK9 inhibitors, which have been shown to reduce blood lipids while simultaneously inhibiting immune rejection in heart transplantation (139). Additionally, PD-1 has been identified as a key player in both graft rejection and amino acid regulation (155, 156). Several new nanomaterials targeting metabolic pathways are being developed to address post-transplant complications, and innovative 3D printing materials are emerging as potential tools in the management of graft rejection (221). Furthermore, the role of microRNAs in MetS and graft rejection represents an exciting avenue for future research. Therapeutic strategies aimed at balancing bile acids and amino acids to alleviate systemic stress are still under investigation, and their potential applications could offer significant benefits for transplant patients in the future.

## References

[1] Neeland IJ Lim S Tchernof A Gastaldelli A Rangaswami J Ndumele CE. Metabolic syndrome. Nat Rev Dis Primers. (2024) 10:77. doi: 10.1038/s41572-024-00563-5 39420195

[2] Bhat M Usmani SE Azhie A Woo M. Metabolic consequences of solid organ transplantation. Endocr Rev. (2021) 42:171–97. doi: 10.1210/endrev/bnaa030 33247713

[3] Sharif A Baboolal K. Metabolic syndrome and solid-organ transplantation. Am J Transplantation. (2010) 10:12–7. doi: 10.1111/j.1600-6143.2009.02882.x 19958337

[4] Tokarz VL MacDonald PE Klip A. The cell biology of systemic insulin function. J Cell Biol. (2018) 217:2273–89. doi: 10.1083/jcb.201802095 PMC6028526 29622564

[5] Bang JB Oh CK Kim YS Kim SH Yu HC Kim CD. Insulin Secretion and Insulin Resistance Trajectories over 1 Year after Kidney Transplantation: A Multicenter Prospective Cohort Study. Endocrinol Metab (Seoul). (2020) 35:820–9. doi: 10.3803/EnM.2020.743 PMC7803593 33202516

[6] Randle PJ Garland PB Hales CN Newsholme EA. The glucose fatty-acid cycle. Its role in insulin sensitivity and the metabolic disturbances of diabetes mellitus. Lancet. (1963) 1:785–9. doi: 10.1016/S0140-6736(63)91500-9 13990765

[7] Boden G Shulman GI. Free fatty acids in obesity and type 2 diabetes: defining their role in the development of insulin resistance and beta-cell dysfunction. Eur J Clin Invest. (2002) 32 Suppl 3:14–23. doi: 10.1046/j.1365-2362.32.s3.3.x 12028371

[8] Okada T Kawano Y Sakakibara T Hazeki O Ui M. Essential role of phosphatidylinositol 3-kinase in insulin-induced glucose transport and antilipolysis in rat adipocytes. Studies with a selective inhibitor wortmannin. J Biol Chem. (1994) 269:3568–73. doi: 10.1016/S0021-9258(17)41901-6 8106400

[9] Murakami T Michelagnoli S Longhi R GianFranceschi G Pazzucconi F Calabresi L. Triglycerides are major determinants of cholesterol esterification/transfer and HDL remodeling in human plasma. Arterioscler Thromb Vasc Biol. (1995) 15:1819–28. doi: 10.1161/01.ATV.15.11.1819 7583561

[10] Tabas I Bornfeldt KE. Macrophage phenotype and function in different stages of atherosclerosis. Circ Res. (2016) 118:653–67. doi: 10.1161/CIRCRESAHA.115.306256 PMC4762068 26892964

[11] Tripathy D Mohanty P Dhindsa S Syed T Ghanim H Aljada A. Elevation of free fatty acids induces inflammation and impairs vascular reactivity in healthy subjects. Diabetes. (2003) 52:2882–7. doi: 10.2337/diabetes.52.12.2882 14633847

[12] Juhan-Vague I Alessi MC Mavri A Morange PE. Plasminogen activator inhibitor-1, inflammation, obesity, insulin resistance and vascular risk. J Thromb Haemost. (2003) 1:1575–9. doi: 10.1046/j.1538-7836.2003.00279.x 12871293

[13] Villarroya F Cereijo R Villarroya J Giralt M. Brown adipose tissue as a secretory organ. Nat Rev Endocrinol. (2017) 13:26–35. doi: 10.1038/nrendo.2016.136 27616452

[14] Berglund ED Vianna CR Donato J Jr. Kim MH Chuang JC Lee CE. Direct leptin action on POMC neurons regulates glucose homeostasis and hepatic insulin sensitivity in mice. J Clin Invest. (2012) 122:1000–9. doi: 10.1172/JCI59816 PMC3287225 22326958

[15] Lord GM Matarese G Howard JK Baker RJ Bloom SR Lechler RI. Leptin modulates the T-cell immune response and reverses starvation-induced immunosuppression. Nature. (1998) 394:897–901. doi: 10.1038/29795 9732873

[16] Patel SB Reams GP Spear RM Freeman RH Villarreal D. Leptin: linking obesity, the metabolic syndrome, and cardiovascular disease. Curr Hypertens Rep. (2008) 10:131–7. doi: 10.1007/s11906-008-0025-y 18474180

[17] Straub LG Scherer PE. Metabolic messengers: adiponectin. Nat Metab. (2019) 1:334–9. doi: 10.1038/s42255-019-0041-z PMC7357716 32661510

[18] Kondo H Shimomura I Matsukawa Y Kumada M Takahashi M Matsuda M. Association of adiponectin mutation with type 2 diabetes: a candidate gene for the insulin resistance syndrome. Diabetes. (2002) 51:2325–8. doi: 10.2337/diabetes.51.7.2325 12086969

[19] Tan L Lu X Danser AHJ Verdonk K. The role of chemerin in metabolic and cardiovascular disease: A literature review of its physiology and pathology from a nutritional perspective. Nutrients. (2023) 15(13):2878. doi: 10.3390/nu15132878 37447205 PMC10343651

[20] Jialal I Devaraj S Kaur H Adams-Huet B Bremer AA. Increased chemerin and decreased omentin-1 in both adipose tissue and plasma in nascent metabolic syndrome. J Clin Endocrinol Metab. (2013) 98:E514–7. doi: 10.1210/jc.2012-3673 23303213

[21] Wang D Yuan GY Wang XZ Jia J Di LL Yang L. Plasma chemerin level in metabolic syndrome. Genet Mol Res. (2013) 12:5986–91. doi: 10.4238/2013.November.26.8 24338392

[22] Dong B Ji W Zhang Y. Elevated serum chemerin levels are associated with the presence of coronary artery disease in patients with metabolic syndrome. Intern Med. (2011) 50:1093–7. doi: 10.2169/internalmedicine.50.5025 21576834

[23] Hauner H. Secretory factors from human adipose tissue and their functional role. Proc Nutr Soc. (2005) 64:163–9. doi: 10.1079/PNS2005428 15960861

[24] Hunter CA Jones SA. IL-6 as a keystone cytokine in health and disease. Nat Immunol. (2015) 16:448–57. doi: 10.1038/ni.3153 25898198

[25] Kraakman MJ Kammoun HL Allen TL Deswaerte V Henstridge DC Estevez E. Blocking IL-6 trans-signaling prevents high-fat diet-induced adipose tissue macrophage recruitment but does not improve insulin resistance. Cell Metab. (2015) 21:403–16. doi: 10.1016/j.cmet.2015.02.006 25738456

[26] Bethin KE Vogt SK Muglia LJ. Interleukin-6 is an essential, corticotropin-releasing hormone-independent stimulator of the adrenal axis during immune system activation. Proc Natl Acad Sci U S A. (2000) 97:9317–22. doi: 10.1073/pnas.97.16.9317 PMC16865 10922080

[27] Tyrrell DJ Goldstein DR. Ageing and atherosclerosis: vascular intrinsic and extrinsic factors and potential role of IL-6. Nat Rev Cardiol. (2021) 18:58–68. doi: 10.1038/s41569-020-0431-7 32918047 PMC7484613

[28] Weisberg SP McCann D Desai M Rosenbaum M Leibel RL Ferrante AW Jr. Obesity is associated with macrophage accumulation in adipose tissue. J Clin Invest. (2003) 112:1796–808. doi: 10.1172/JCI200319246 PMC296995 14679176

[29] Hotamisligil GS Arner P Caro JF Atkinson RL Spiegelman BM. Increased adipose tissue expression of tumor necrosis factor-alpha in human obesity and insulin resistance. J Clin Invest. (1995) 95:2409–15. doi: 10.1172/JCI117936 PMC295872 7738205

[30] Cai D Yuan M Frantz DF Melendez PA Hansen L Lee J. Local and systemic insulin resistance resulting from hepatic activation of IKK-beta and NF-kappaB. Nat Med. (2005) 11:183–90. doi: 10.1038/nm1166 PMC1440292 15685173

[31] Hirosumi J Tuncman G Chang L Görgün CZ Uysal KT Maeda K. A central role for JNK in obesity and insulin resistance. Nature. (2002) 420:333–6. doi: 10.1038/nature01137 12447443

[32] Hotamisligil GS Murray DL Choy LN Spiegelman BM. Tumor necrosis factor alpha inhibits signaling from the insulin receptor. Proc Natl Acad Sci U S A. (1994) 91:4854–8. doi: 10.1073/pnas.91.11.4854 PMC43887 8197147

[33] Zhang HH Halbleib M Ahmad F Manganiello VC Greenberg AS. Tumor necrosis factor-alpha stimulates lipolysis in differentiated human adipocytes through activation of extracellular signal-related kinase and elevation of intracellular cAMP. Diabetes. (2002) 51:2929–35. doi: 10.2337/diabetes.51.10.2929 12351429

[34] Mori K Emoto M Inaba M. Fetuin-A: a multifunctional protein. Recent Pat Endocr Metab Immune Drug Discov. (2011) 5:124–46. doi: 10.2174/187221411799015372 22074587

[35] Jialal I Devaraj S Bettaieb A Haj F Adams-Huet B. Increased adipose tissue secretion of Fetuin-A, lipopolysaccharide-binding protein and high-mobility group box protein 1 in metabolic syndrome. Atherosclerosis. (2015) 241:130–7. doi: 10.1016/j.atherosclerosis.2015.04.814 25978344

[36] Pérez-Sotelo D Roca-Rivada A Larrosa-García M Castelao C Baamonde I Baltar J. Visceral and subcutaneous adipose tissue express and secrete functional alpha2hsglycoprotein (fetuin a) especially in obesity. Endocrine. (2017) 55:435–46. doi: 10.1007/s12020-016-1132-1 27738888

[37] Bourebaba L Marycz K. Pathophysiological implication of fetuin-A glycoprotein in the development of metabolic disorders: A concise review. J Clin Med. (2019) 8(12):2033. doi: 10.3390/jcm8122033 31766373 PMC6947209

[38] Petruzzelli M Moschetta A. Intestinal ecology in the metabolic syndrome. Cell Metab. (2010) 11:345–6. doi: 10.1016/j.cmet.2010.04.012 20444415

[39] Zhang Z Mocanu V Deehan EC Hotte N Zhu Y Wei S. Recipient microbiome-related features predicting metabolic improvement following fecal microbiota transplantation in adults with severe obesity and metabolic syndrome: a secondary analysis of a phase 2 clinical trial. Gut Microbes. (2024) 16:2345134. doi: 10.1080/19490976.2024.2345134 38685731 PMC11062372

[40] Chassaing B Gewirtz AT. Gut microbiota, low-grade inflammation, and metabolic syndrome. Toxicol Pathol. (2014) 42:49–53. doi: 10.1177/0192623313508481 24285672

[41] Pradhan AD Manson JE Rifai N Buring JE Ridker PM. C-reactive protein, interleukin 6, and risk of developing type 2 diabetes mellitus. Jama. (2001) 286:327–34. doi: 10.1001/jama.286.3.327 11466099

[42] Hotamisligil GS. Inflammation, metaflammation and immunometabolic disorders. Nature. (2017) 542:177–85. doi: 10.1038/nature21363 28179656

[43] O’Neill LA Golenbock D Bowie AG. The history of Toll-like receptors - redefining innate immunity. Nat Rev Immunol. (2013) 13:453–60. doi: 10.1038/nri3446 23681101

[44] Yoshida N Emoto T Yamashita T Watanabe H Hayashi T Tabata T. Bacteroides vulgatus and Bacteroides dorei Reduce Gut Microbial Lipopolysaccharide Production and Inhibit Atherosclerosis. Circulation. (2018) 138:2486–98. doi: 10.1161/CIRCULATIONAHA.118.033714 30571343

[45] Molinaro A Wahlström A Marschall HU. Role of bile acids in metabolic control. Trends Endocrinol Metab. (2018) 29:31–41. doi: 10.1016/j.tem.2017.11.002 29195686

[46] Thomas C Pellicciari R Pruzanski M Auwerx J Schoonjans K. Targeting bile-acid signalling for metabolic diseases. Nat Rev Drug Discov. (2008) 7:678–93. doi: 10.1038/nrd2619 18670431

[47] Sayin SI Wahlström A Felin J Jäntti S Marschall HU Bamberg K. Gut microbiota regulates bile acid metabolism by reducing the levels of tauro-beta-muricholic acid, a naturally occurring FXR antagonist. Cell Metab. (2013) 17:225–35. doi: 10.1016/j.cmet.2013.01.003 23395169

[48] Jones BV Begley M Hill C Gahan CG Marchesi JR. Functional and comparative metagenomic analysis of bile salt hydrolase activity in the human gut microbiome. Proc Natl Acad Sci U S A. (2008) 105:13580–5. doi: 10.1073/pnas.0804437105 PMC2533232 18757757

[49] Wahlström A Sayin SI Marschall HU Bäckhed F. Intestinal crosstalk between bile acids and microbiota and its impact on host metabolism. Cell Metab. (2016) 24:41–50. doi: 10.1016/j.cmet.2016.05.005 27320064

[50] David LA Maurice CF Carmody RN Gootenberg DB Button JE Wolfe BE. Diet rapidly and reproducibly alters the human gut microbiome. Nature. (2014) 505:559–63. doi: 10.1038/nature12820 PMC3957428 24336217

[51] Trabelsi MS Daoudi M Prawitt J Ducastel S Touche V Sayin SI. Farnesoid X receptor inhibits glucagon-like peptide-1 production by enteroendocrine L cells. Nat Commun. (2015) 6:7629. doi: 10.1038/ncomms8629 26134028 PMC4579574

[52] Warden BA Duell PB. Management of dyslipidemia in adult solid organ transplant recipients. J Clin Lipidol. (2019) 13:231–45. doi: 10.1016/j.jacl.2019.01.011 30928441

[53] Wissing KM Pipeleers L. Obesity, metabolic syndrome and diabetes mellitus after renal transplantation: prevention and treatment. Transplant Rev (Orlando). (2014) 28:37–46. doi: 10.1016/j.trre.2013.12.004 24507957

[54] Piotti G Gandolfini I Palmisano A Maggiore U. Metabolic risk profile in kidney transplant candidates and recipients. Nephrol Dial Transplant. (2019) 34:388–400. doi: 10.1093/ndt/gfy151 29800310

[55] Alfieri C Campioli E Fiorina P Orsi E Grancini V Regalia A. Post-transplant diabetes mellitus in kidney-transplanted patients: related factors and impact on long-term outcome. Nutrients. (2024) 16(10):1520. doi: 10.3390/nu16101520 38794758 PMC11123789

[56] D’Elia JA Weinrauch LA. Hyperglycemia and hyperlipidemia with kidney or liver transplantation: A review. Biol (Basel). (2023) 12(9):1185. doi: 10.3390/biology12091185 PMC10525610 37759585

[57] Ling Q Xu X Wang K Wang C Xiang P Zhang X. Donor PPARα Gene polymorphisms influence the susceptibility to glucose and lipid disorders in liver transplant recipients: A strobe-compliant observational study. Med (Baltimore). (2015) 94:e1421. doi: 10.1097/MD.0000000000001421 PMC4616503 26334901

[58] Dalla Via V Halter JP Gerull S Arranto C Tichelli A Heim D. New-onset post-transplant diabetes and therapy in long-term survivors after allogeneic hematopoietic stem cell transplantation. In Vivo. (2020) 34:3545–9. doi: 10.21873/invivo.12197 PMC7811658 33144466

[59] Lindner S Miltiadous O Ramos RJF Paredes J Kousa AI Dai A. Altered microbial bile acid metabolism exacerbates T cell-driven inflammation during graft-versus-host disease. Nat Microbiol. (2024) 9:614–30. doi: 10.1038/s41564-024-01617-w PMC11196888 38429422

[60] Watt KD Charlton MR. Metabolic syndrome and liver transplantation: a review and guide to management. J Hepatol. (2010) 53:199–206. doi: 10.1016/j.jhep.2010.01.040 20451282

[61] Zoccali C Postorino M Marino C Pizzini P Cutrupi S Tripepi G. Waist circumference modifies the relationship between the adipose tissue cytokines leptin and adiponectin and all-cause and cardiovascular mortality in haemodialysis patients. J Intern Med. (2011) 269:172–81. doi: 10.1111/j.1365-2796.2010.02288.x 21138492

[62] Abdelrahman SM Samir B Alazem EAA Musa N. Effect of pre and post-transplant body mass index on pediatric kidney transplant outcomes. BMC Pediatr. (2022) 22:299. doi: 10.1186/s12887-022-03344-9 35597898 PMC9123701

[63] LeGrand EK. Beyond nutritional immunity: immune-stressing challenges basic paradigms of immunometabolism and immunology. Front Nutr. (2025) 12:1508767. doi: 10.3389/fnut.2025.1508767 40013164 PMC11860096

[64] Muri J Kopf M. Redox regulation of immunometabolism. Nat Rev Immunol. (2021) 21:363–81. doi: 10.1038/s41577-020-00478-8 33340021

[65] Porrini E Delgado P Bigo C Alvarez A Cobo M Checa MD. Impact of metabolic syndrome on graft function and survival after cadaveric renal transplantation. Am J Kidney Dis. (2006) 48:134–42. doi: 10.1053/j.ajkd.2006.04.078 16797396

[66] Cordero Fort A Gavira JJ Alegría-Barrero E Castaño S Martín A Ubilla M. Prevalence of metabolic syndrome in heart transplant patients: role of previous cardiopathy and years since the procedure–the TRACA study. J Heart Lung Transplant. (2006) 25:1192–8. doi: 10.1016/j.healun.2006.06.012 17045931

[67] Biadi O Potena L Fearon WF Luikart HI Yeung A Ferrara R. Interplay between systemic inflammation and markers of insulin resistance in cardiovascular prognosis after heart transplantation. J Heart Lung Transplant. (2007) 26:324–30. doi: 10.1016/j.healun.2007.01.020 17403472

[68] Sánchez-Gómez JM Martínez-Dolz L Sánchez-Lázaro I Almenar L Sánchez-Lacuesta E Muñoz-Giner B. Influence of metabolic syndrome on development of cardiac allograft vasculopathy in the transplanted heart. Transplantation. (2012) 93:106–11. doi: 10.1097/TP.0b013e3182398058 22134367

[69] Baldwin WM 3rd Halushka MK Valujskikh A Fairchild RL. B cells in cardiac transplants: from clinical questions to experimental models. Semin Immunol. (2012) 24:122–30. doi: 10.1016/j.smim.2011.08.017 PMC3307908 21937238

[70] Mikolasevic I Orlic L Hrstic I Milic S. Metabolic syndrome and non-alcoholic fatty liver disease after liver or kidney transplantation. Hepatol Res. (2016) 46:841–52. doi: 10.1111/hepr.12642 26713425

[71] Schneider JG Finck BN Ren J Standley KN Takagi M Maclean KH. ATM-dependent suppression of stress signaling reduces vascular disease in metabolic syndrome. Cell Metab. (2006) 4:377–89. doi: 10.1016/j.cmet.2006.10.002 17084711

[72] Shivaswamy V Boerner B Larsen J. Post-transplant diabetes mellitus: causes, treatment, and impact on outcomes. Endocr Rev. (2016) 37:37–61. doi: 10.1210/er.2015-1084 26650437 PMC4740345

[73] Kavazović I Krapić M Beumer-Chuwonpad A Polić B Turk Wensveen T Lemmermann NA. Hyperglycemia and not hyperinsulinemia mediates diabetes-induced memory CD8 T-cell dysfunction. Diabetes. (2022) 71:706–21. doi: 10.2337/db21-0209 35044446

[74] Jacobs SR Herman CE Maciver NJ Wofford JA Wieman HL Hammen JJ. Glucose uptake is limiting in T cell activation and requires CD28-mediated Akt-dependent and independent pathways. J Immunol. (2008) 180:4476–86. doi: 10.4049/jimmunol.180.7.4476 PMC2593791 18354169

[75] Šestan M Marinović S Kavazović I Cekinović Đ Wueest S Turk Wensveen T. Virus-induced interferon-γ Causes insulin resistance in skeletal muscle and derails glycemic control in obesity. Immunity. (2018) 49:164–77.e6. doi: 10.1016/j.immuni.2018.05.005 29958802

[76] Tsai S Clemente-Casares X Zhou AC Lei H Ahn JJ Chan YT. Insulin receptor-mediated stimulation boosts T cell immunity during inflammation and infection. Cell Metab. (2018) 28:922–34.e4. doi: 10.1016/j.cmet.2018.08.003 30174303

[77] Martinez N Vallerskog T West K Nunes-Alves C Lee J Martens GW. Chromatin decondensation and T cell hyperresponsiveness in diabetes-associated hyperglycemia. J Immunol. (2014) 193:4457–68. doi: 10.4049/jimmunol.1401125 PMC4242014 25246495

[78] Pham TD Chng MHY Roskin KM Jackson KJL Nguyen KD Glanville J. High-fat diet induces systemic B-cell repertoire changes associated with insulin resistance. Mucosal Immunol. (2017) 10:1468–79. doi: 10.1038/mi.2017.25 28422186

[79] Zhai X Qian G Wang Y Chen X Lu J Zhang Y. Elevated B cell activation is associated with type 2 diabetes development in obese subjects. Cell Physiol Biochem. (2016) 38:1257–66. doi: 10.1159/000443073 26982979

[80] Liu B Hu Y Wu Q Zeng Y Xiao Y Zeng X. Qualitative and quantitative analysis of B-cell-produced antibodies in vitreous humor of type 2 diabetic patients with diabetic retinopathy. J Diabetes Res. (2020) 2020:4631290. doi: 10.1155/2020/4631290 32714992 PMC7352131

[81] Pal A Lin CT Boykov I Benson E Kidd G Fisher-Wellman KH. High fat diet-induced obesity dysregulates splenic B cell mitochondrial activity. Nutrients. (2023) 15(22):4807. doi: 10.3390/nu15224807 38004202 PMC10675399

[82] Edgar L Akbar N Braithwaite AT Krausgruber T Gallart-Ayala H Bailey J. Hyperglycemia induces trained immunity in macrophages and their precursors and promotes atherosclerosis. Circulation. (2021) 144:961–82. doi: 10.1161/CIRCULATIONAHA.120.046464 PMC8448412 34255973

[83] Torres-Castro I Arroyo-Camarena ÚD Martínez-Reyes CP Gómez-Arauz AY Dueñas-Andrade Y Hernández-Ruiz J. Human monocytes and macrophages undergo M1-type inflammatory polarization in response to high levels of glucose. Immunol Lett. (2016) 176:81–9. doi: 10.1016/j.imlet.2016.06.001 27269375

[84] Khan MA Schultz S Othman A Fleming T Lebrón-Galán R Rades D. Hyperglycemia in stroke impairs polarization of monocytes/macrophages to a protective noninflammatory cell type. J Neurosci. (2016) 36:9313–25. doi: 10.1523/JNEUROSCI.0473-16.2016 PMC6601875 27605608

[85] Thiem K Keating ST Netea MG Riksen NP Tack CJ van Diepen J. Hyperglycemic memory of innate immune cells promotes *in vitro* proinflammatory responses of human monocytes and murine macrophages. J Immunol. (2021) 206:807–13. doi: 10.4049/jimmunol.1901348 33431659

[86] Mossel DM Moganti K Riabov V Weiss C Kopf S Cordero J. Epigenetic regulation of S100A9 and S100A12 expression in monocyte-macrophage system in hyperglycemic conditions. Front Immunol. (2020) 11:1071. doi: 10.3389/fimmu.2020.01071 32582175 PMC7280556

[87] Zhang B Yang Y Yi J Zhao Z Ye R. Hyperglycemia modulates M1/M2 macrophage polarization via reactive oxygen species overproduction in ligature-induced periodontitis. J Periodontal Res. (2021) 56:991–1005. doi: 10.1111/jre.12912 34190354

[88] Sousa ESA Queiroz LAD Guimarães JPT Pantoja KC Barros RS Epiphanio S. The influence of high glucose conditions on macrophages and its effect on the autophagy pathway. Front Immunol. (2023) 14:1130662. doi: 10.3389/fimmu.2023.1130662 37122742 PMC10130370

[89] Matuschik L Riabov V Schmuttermaier C Sevastyanova T Weiss C Klüter H. Hyperglycemia induces inflammatory response of human macrophages to CD163-mediated scavenging of hemoglobin-haptoglobin complexes. Int J Mol Sci. (2022) 23(3):1385. doi: 10.3390/ijms23031385 35163309 PMC8836198

[90] Rao Z Sun J Pan X Chen Z Sun H Zhang P. Hyperglycemia aggravates hepatic ischemia and reperfusion injury by inhibiting liver-resident macrophage M2 polarization via C/EBP homologous protein-mediated endoplasmic reticulum stress. Front Immunol. (2017) 8:1299. doi: 10.3389/fimmu.2017.01299 29081777 PMC5645540

[91] Toda G Soeda K Okazaki Y Kobayashi N Masuda Y Arakawa N. Insulin- and lipopolysaccharide-mediated signaling in adipose tissue macrophages regulates postprandial glycemia through akt-mTOR activation. Mol Cell. (2020) 79:43–53.e4. doi: 10.1016/j.molcel.2020.04.033 32464093 PMC11969070

[92] Hu N Zhang X Zhang X Guan Y He R Xue E. Inhibition of Notch activity suppresses hyperglycemia-augmented polarization of macrophages to the M1 phenotype and alleviates acute pancreatitis. Clin Sci (Lond). (2022) 136:455–71. doi: 10.1042/CS20211031 PMC8987789 35302580

[93] Cheng CI Chen PH Lin YC Kao YH. High glucose activates Raw264.7 macrophages through RhoA kinase-mediated signaling pathway. Cell Signal. (2015) 27:283–92. doi: 10.1016/j.cellsig.2014.11.012 25446262

[94] Morey M O’Gaora P Pandit A Hélary C. Hyperglycemia acts in synergy with hypoxia to maintain the pro-inflammatory phenotype of macrophages. PloS One. (2019) 14:e0220577. doi: 10.1371/journal.pone.0220577 31415598 PMC6695165

[95] Chung S Ranjan R Lee YG Park GY Karpurapu M Deng J. Distinct role of FoxO1 in M-CSF- and GM-CSF-differentiated macrophages contributes LPS-mediated IL-10: implication in hyperglycemia. J Leukoc Biol. (2015) 97:327–39. doi: 10.1189/jlb.3A0514-251R PMC4304426 25420919

[96] Moganti K Li F Schmuttermaier C Riemann S Klüter H Gratchev A. Hyperglycemia induces mixed M1/M2 cytokine profile in primary human monocyte-derived macrophages. Immunobiology. (2017) 222:952–9. doi: 10.1016/j.imbio.2016.07.006 27492721

[97] Zhao Z Ming Y Li X Tan H He X Yang L. Hyperglycemia aggravates periodontitis via autophagy impairment and ROS-inflammasome-mediated macrophage pyroptosis. Int J Mol Sci. (2023) 24(7):6309. doi: 10.3390/ijms24076309 37047282 PMC10094233

[98] Zhang P Wang Q Nie L Zhu R Zhou X Zhao P. Hyperglycemia-induced inflamm-aging accelerates gingival senescence via NLRC4 phosphorylation. J Biol Chem. (2019) 294:18807–19. doi: 10.1074/jbc.RA119.010648 PMC6901307 31676687

[99] Johnson CD Fischer D Smith IM Aranda-Espinoza H Fisher JP. Hyperglycemic conditions enhance the mechanosensitivity of proinflammatory RAW264.7 macrophages. Tissue Eng Part A. (2023) 29:172–84. doi: 10.1089/ten.tea.2022.0151 PMC10024269 36517975

[100] Aguilar-Ballester M Herrero-Cervera A Vinué Á Martínez-Hervás S González-Navarro H. Impact of cholesterol metabolism in immune cell function and atherosclerosis. Nutrients. (2020) 12(7):2021. doi: 10.3390/nu12072021 32645995 PMC7400846

[101] Bensinger SJ Bradley MN Joseph SB Zelcer N Janssen EM Hausner MA. LXR signaling couples sterol metabolism to proliferation in the acquired immune response. Cell. (2008) 134:97–111. doi: 10.1016/j.cell.2008.04.052 18614014 PMC2626438

[102] Bietz A Zhu H Xue M Xu C. Cholesterol metabolism in T cells. Front Immunol. (2017) 8:1664. doi: 10.3389/fimmu.2017.01664 29230226 PMC5711771

[103] Shimano H Sato R. SREBP-regulated lipid metabolism: convergent physiology - divergent pathophysiology. Nat Rev Endocrinol. (2017) 13:710–30. doi: 10.1038/nrendo.2017.91 28849786

[104] Miura K Yu R Entwistle TR McKenzie SC Green AC. Long-term changes in body weight and serum cholesterol in heart transplant recipients. Clin Transplant. (2022) 36:e14819. doi: 10.1111/ctr.v36.12 36074751 PMC10909516

[105] Garthwaite EA Will EJ Bartlett C Richardson D Newstead CG. Patient-specific prompts in the cholesterol management of renal transplant outpatients: results and analysis of underperformance. Transplantation. (2004) 78:1042–7. doi: 10.1097/01.TP.0000137340.22880.C8 15480172

[106] Mailer RKW Gisterå A Polyzos KA Ketelhuth DFJ Hansson GK. Hypercholesterolemia enhances T cell receptor signaling and increases the regulatory T cell population. Sci Rep. (2017) 7:15655. doi: 10.1038/s41598-017-15546-8 29142309 PMC5688061

[107] Proto JD Doran AC Subramanian M Wang H Zhang M Sozen E. Hypercholesterolemia induces T cell expansion in humanized immune mice. J Clin Invest. (2018) 128:2370–5. doi: 10.1172/JCI97785 PMC5983331 29708512

[108] Chyu KY Lio WM Dimayuga PC Zhou J Zhao X Yano J. Cholesterol lowering modulates T cell function *in vivo* and *in vitro* . PloS One. (2014) 9:e92095. doi: 10.1371/journal.pone.0092095 24647529 PMC3960213

[109] Zhou X Johnston TP Johansson D Parini P Funa K Svensson J. Hypercholesterolemia leads to elevated TGF-beta1 activity and T helper 3-dependent autoimmune responses in atherosclerotic mice. Atherosclerosis. (2009) 204:381–7. doi: 10.1016/j.atherosclerosis.2008.10.017 19054515

[110] Mailer RKW Gisterå A Polyzos KA Ketelhuth DFJ Hansson GK. Hypercholesterolemia induces differentiation of regulatory T cells in the liver. Circ Res. (2017) 120:1740–53. doi: 10.1161/CIRCRESAHA.116.310054 28420668

[111] Mercadante ER Lorenz UM. Breaking free of control: how conventional T cells overcome regulatory T cell suppression. Front Immunol. (2016) 7:193. doi: 10.3389/fimmu.2016.00193 27242798 PMC4870238

[112] Klingenberg R Gerdes N Badeau RM Gisterå A Strodthoff D Ketelhuth DF. Depletion of FOXP3+ regulatory T cells promotes hypercholesterolemia and atherosclerosis. J Clin Invest. (2013) 123:1323–34. doi: 10.1172/JCI63891 PMC3582120 23426179

[113] Zhang H Xia N Tang T Nie S Zha L Zhang M. Cholesterol suppresses human iTreg differentiation and nTreg function through mitochondria-related mechanisms. J Transl Med. (2023) 21:224. doi: 10.1186/s12967-023-03896-z 36973679 PMC10045251

[114] Hsieh V Kim MJ Gelissen IC Brown AJ Sandoval C Hallab JC. Cellular cholesterol regulates ubiquitination and degradation of the cholesterol export proteins ABCA1 and ABCG1. J Biol Chem. (2014) 289:7524–36. doi: 10.1074/jbc.M113.515890 PMC3953266 24500716

[115] Wen X Zhao WH Chen LZ Qu W Liu HX Yan HY. Attenuated cholesterol metabolism pathway suppresses regulatory T cell development in prenatal nicotine exposed female mice. Toxicology. (2019) 428:152309. doi: 10.1016/j.tox.2019.152309 31629012

[116] Cheng HY Gaddis DE Wu R McSkimming C Haynes LD Taylor AM. Loss of ABCG1 influences regulatory T cell differentiation and atherosclerosis. J Clin Invest. (2016) 126:3236–46. doi: 10.1172/JCI83136 PMC5004951 27482882

[117] Armstrong AJ Gebre AK Parks JS Hedrick CC. ATP-binding cassette transporter G1 negatively regulates thymocyte and peripheral lymphocyte proliferation. J Immunol. (2010) 184:173–83. doi: 10.4049/jimmunol.0902372 PMC3316475 19949102

[118] Waddington KE Robinson GA Rubio-Cuesta B Chrifi-Alaoui E Andreone S Poon KS. LXR directly regulates glycosphingolipid synthesis and affects human CD4+ T cell function. Proc Natl Acad Sci U S A. (2021) 118(21):e2017394118. doi: 10.1073/pnas.2017394118 34006637 PMC8166169

[119] Cui G Qin X Wu L Zhang Y Sheng X Yu Q. Liver X receptor (LXR) mediates negative regulation of mouse and human Th17 differentiation. J Clin Invest. (2011) 121:658–70. doi: 10.1172/JCI42974 PMC3026720 21266776

[120] Xu J Wagoner G Douglas JC Drew PD. Liver X receptor agonist regulation of Th17 lymphocyte function in autoimmunity. J Leukoc Biol. (2009) 86:401–9. doi: 10.1189/jlb.1008600 PMC2726767 19406833

[121] Kim J Lee H Lee JE Choi G Chung H Kim D. Liver X receptor controls follicular helper T cell differentiation via repression of TCF-1. Proc Natl Acad Sci U S A. (2023) 120:e2213793120. doi: 10.1073/pnas.2213793120 36802434 PMC9992818

[122] Tan S Feng X Liu Z Wang Q Jiang Q Ye X. The pro-inflammatory effect of triglyceride on human CD4(+) T cells and experimental autoimmune uveitis. Clin Immunol. (2022) 240:109056. doi: 10.1016/j.clim.2022.109056 35659924

[123] van Os BW Vos WG Bosmans LA van Tiel CM Lith SC den Toom MS. Hyperlipidaemia elicits an atypical, T helper 1-like CD4(+) T-cell response: a key role for very low-density lipoprotein. Eur Heart J Open. (2023) 3:oead013. doi: 10.1093/ehjopen/oead013 36969380 PMC10032356

[124] Trindade BC Ceglia S Berthelette A Raso F Howley K Muppidi JR. The cholesterol metabolite 25-hydroxycholesterol restrains the transcriptional regulator SREBP2 and limits intestinal IgA plasma cell differentiation. Immunity. (2021) 54:2273–87.e6. doi: 10.1016/j.immuni.2021.09.004 34644558 PMC8570345

[125] Schmitz T Freuer D Linseisen J Meisinger C. Associations between serum cholesterol and immunophenotypical characteristics of circulatory B cells and Tregs. J Lipid Res. (2023) 64:100399. doi: 10.1016/j.jlr.2023.100399 37276940 PMC10394386

[126] Burger F Miteva K Baptista D Roth A Fraga-Silva RA Martel C. Follicular regulatory helper T cells control the response of regulatory B cells to a high-cholesterol diet. Cardiovasc Res. (2021) 117:743–55. doi: 10.1093/cvr/cvaa069 PMC7898950 32219371

[127] Ito A Hong C Oka K Salazar JV Diehl C Witztum JL. Cholesterol accumulation in CD11c(+) immune cells is a causal and targetable factor in autoimmune disease. Immunity. (2016) 45:1311–26. doi: 10.1016/j.immuni.2016.11.008 PMC5181791 28002731

[128] Gomes AL Carvalho T Serpa J Torre C Dias S. Hypercholesterolemia promotes bone marrow cell mobilization by perturbing the SDF-1:CXCR4 axis. Blood. (2010) 115:3886–94. doi: 10.1182/blood-2009-08-240580 20009035

[129] Higgins CB Adams JA Ward MH Greenberg ZJ Milewska M Sun J. The tetraspanin transmembrane protein CD53 mediates dyslipidemia and integrates inflammatory and metabolic signaling in hepatocytes. J Biol Chem. (2023) 299:102835. doi: 10.1016/j.jbc.2022.102835 36581203 PMC9900517

[130] Qin WH Yang ZS Li M Chen Y Zhao XF Qin YY. High serum levels of cholesterol increase antitumor functions of nature killer cells and reduce growth of liver tumors in mice. Gastroenterology. (2020) 158:1713–27. doi: 10.1053/j.gastro.2020.01.028 31972238

[131] Jiao D Sun R Ren X Wang Y Tian P Wang Y. Lipid accumulation-mediated histone hypoacetylation drives persistent NK cell dysfunction in anti-tumor immunity. Cell Rep. (2023) 42:113211. doi: 10.1016/j.celrep.2023.113211 37792534

[132] Hu X Jia X Xu C Wei Y Wang Z Liu G. Downregulation of NK cell activities in Apolipoprotein C-III-induced hyperlipidemia resulting from lipid-induced metabolic reprogramming and crosstalk with lipid-laden dendritic cells. Metabolism. (2021) 120:154800. doi: 10.1016/j.metabol.2021.154800 34051224

[133] Ryu H Kim J Kim D Lee JE Chung Y. Cellular and molecular links between autoimmunity and lipid metabolism. Mol Cells. (2019) 42:747–54. doi: 10.14348/molcells.2019.0196 PMC6883973 31766832

[134] Krauzová E Kračmerová J Rossmeislová L Mališová L Tencerová M Koc M. Acute hyperlipidemia initiates proinflammatory and proatherogenic changes in circulation and adipose tissue in obese women. Atherosclerosis. (2016) 250:151–7. doi: 10.1016/j.atherosclerosis.2016.04.021 27236705

[135] Ray AG Choudhury KR Chakraborty S Chakravarty D Chander V Jana B. Novel mechanism of cholesterol transport by ABCA5 in macrophages and its role in dyslipidemia. J Mol Biol. (2020) 432:4922–41. doi: 10.1016/j.jmb.2020.07.006 32687853

[136] Li Q Park K Xia Y Matsumoto M Qi W Fu J. Regulation of macrophage apoptosis and atherosclerosis by lipid-induced PKCδ Isoform activation. Circ Res. (2017) 121:1153–67. doi: 10.1161/CIRCRESAHA.117.311606 PMC6176491 28855204

[137] Chaib M Holt JR Fisher EL Sipe LM Bohm MS Joseph SC. Protein kinase C delta regulates mononuclear phagocytes and hinders response to immunotherapy in cancer. Sci Adv. (2023) 9:eadd3231. doi: 10.1126/sciadv.add3231 38134280 PMC10745701

[138] Wang Y Zou Y Jiang Q Li W Chai X Zhao T. Ox-LDL-induced CD80(+) macrophages expand pro-atherosclerotic NKT cells via CD1d in atherosclerotic mice and hyperlipidemic patients. Am J Physiol Cell Physiol. (2024) 326:C1563–c72. doi: 10.1152/ajpcell.00043.2024 38586879

[139] Zhang X Xu H Yu J Cui J Chen Z Li Y. Immune regulation of the liver through the PCSK9/CD36 pathway during heart transplant rejection. Circulation. (2023) 148:336–53. doi: 10.1161/CIRCULATIONAHA.123.062788 37232170

[140] Biswas S Gao D Altemus JB Rekhi UR Chang E Febbraio M. Circulating CD36 is increased in hyperlipidemic mice: Cellular sources and triggers of release. Free Radic Biol Med. (2021) 168:180–8. doi: 10.1016/j.freeradbiomed.2021.03.004 PMC8085123 33775772

[141] Phu TA Ng M Vu NK Gao AS Raffai RL. ApoE expression in macrophages communicates immunometabolic signaling that controls hyperlipidemia-driven hematopoiesis & inflammation via extracellular vesicles. J Extracell Vesicles. (2023) 12:e12345. doi: 10.1002/jev2.12345 37593979 PMC10436255

[142] Zhu S Liu Y Xia G Wang X Du A Wu J. Modulation of cardiac resident macrophages immunometabolism upon high-fat-diet feeding in mice. Front Immunol. (2024) 15:1371477. doi: 10.3389/fimmu.2024.1371477 39007149 PMC11239335

[143] Shan B Wang X Wu Y Xu C Xia Z Dai J. The metabolic ER stress sensor IRE1α suppresses alternative activation of macrophages and impairs energy expenditure in obesity. Nat Immunol. (2017) 18:519–29. doi: 10.1038/ni.3709 28346409

[144] Panico C Felicetta A Kunderfranco P Cremonesi M Salvarani N Carullo P. Single-cell RNA sequencing reveals metabolic stress-dependent activation of cardiac macrophages in a model of dyslipidemia-induced diastolic dysfunction. Circulation. (2024) 150:1517–32. doi: 10.1161/CIRCULATIONAHA.122.062984 38126199

[145] Drechsler M Megens RT van Zandvoort M Weber C Soehnlein O. Hyperlipidemia-triggered neutrophilia promotes early atherosclerosis. Circulation. (2010) 122:1837–45. doi: 10.1161/CIRCULATIONAHA.110.961714 20956207

[146] Herz J Sabellek P Lane TE Gunzer M Hermann DM Doeppner TR. Role of neutrophils in exacerbation of brain injury after focal cerebral ischemia in hyperlipidemic mice. Stroke. (2015) 46:2916–25. doi: 10.1161/STROKEAHA.115.010620 PMC4589522 26337969

[147] Fernández-García V González-Ramos S Avendaño-Ortiz J Martín-Sanz P Gómez-Coronado D Delgado C. High-fat diet activates splenic NOD1 and enhances neutrophil recruitment and neutrophil extracellular traps release in the spleen of ApoE-deficient mice. Cell Mol Life Sci. (2022) 79:396. doi: 10.1007/s00018-022-04415-x 35789437 PMC9256580

[148] Yu K Dong Q Mao X Meng K Zhao X Ji Q. Disruption of the TSLP-TSLPR-LAP signaling between epithelial and dendritic cells through hyperlipidemia contributes to regulatory T-Cell defects in atherosclerotic mice. Atherosclerosis. (2015) 238:278–88. doi: 10.1016/j.atherosclerosis.2014.12.019 25544178

[149] Ma Y Ma L Ma J Wu R Zou Y Ge J. Hyperlipidemia inhibits the protective effect of lisinopril after myocardial infarction via activation of dendritic cells. J Cell Mol Med. (2020) 24:4082–91. doi: 10.1111/jcmm.15060 PMC7171409 32073735

[150] Christ A Maas SL Jin H Lu C Legein B Wijnands E. *In situ* lipid-loading activates peripheral dendritic cell subsets characterized by cellular ROS accumulation but compromises their capacity to prime naïve T cells. Free Radic Biol Med. (2024) 210:406–15. doi: 10.1016/j.freeradbiomed.2023.11.044 38061606

[151] Shamshiev AT Ampenberger F Ernst B Rohrer L Marsland BJ Kopf M. Dyslipidemia inhibits Toll-like receptor-induced activation of CD8alpha-negative dendritic cells and protective Th1 type immunity. J Exp Med. (2007) 204:441–52. doi: 10.1084/jem.20061737 PMC2118729 17296788

[152] Packard RR Maganto-García E Gotsman I Tabas I Libby P Lichtman AH. CD11c(+) dendritic cells maintain antigen processing, presentation capabilities, and CD4(+) T-cell priming efficacy under hypercholesterolemic conditions associated with atherosclerosis. Circ Res. (2008) 103:965–73. doi: 10.1161/CIRCRESAHA.108.185793 PMC2668806 18832748

[153] Yang L Chu Z Liu M Zou Q Li J Liu Q. Amino acid metabolism in immune cells: essential regulators of the effector functions, and promising opportunities to enhance cancer immunotherapy. J Hematol Oncol. (2023) 16:59. doi: 10.1186/s13045-023-01453-1 37277776 PMC10240810

[154] Delgoffe GM Pollizzi KN Waickman AT Heikamp E Meyers DJ Horton MR. The kinase mTOR regulates the differentiation of helper T cells through the selective activation of signaling by mTORC1 and mTORC2. Nat Immunol. (2011) 12:295–303. doi: 10.1038/ni.2005 21358638 PMC3077821

[155] Byun JK Park M Lee S Yun JW Lee J Kim JS. Inhibition of glutamine utilization synergizes with immune checkpoint inhibitor to promote antitumor immunity. Mol Cell. (2020) 80:592–606.e8. doi: 10.1016/j.molcel.2020.10.015 33159855

[156] Cui J Xu H Yu J Ran S Zhang X Li Y. Targeted depletion of PD-1-expressing cells induces immune tolerance through peripheral clonal deletion. Sci Immunol. (2024) 9:eadh0085. doi: 10.1126/sciimmunol.adh0085 38669317

[157] Yang B Wang X Ren X. Amino acid metabolism related to immune tolerance by MDSCs. Int Rev Immunol. (2012) 31:177–83. doi: 10.3109/08830185.2012.679989 22587019

[158] Song S Lou Y Mao Y Wen X Fan M He Z. Alteration of gut microbiome and correlated amino acid metabolism contribute to hyperuricemia and th17-driven inflammation in uox-KO mice. Front Immunol. (2022) 13:804306. doi: 10.3389/fimmu.2022.804306 35197978 PMC8858814

[159] Tang K Zhang H Deng J Wang D Liu S Lu S. Ammonia detoxification promotes CD8(+) T cell memory development by urea and citrulline cycles. Nat Immunol. (2023) 24:162–73. doi: 10.1038/s41590-022-01365-1 36471170

[160] Xia X Cao G Sun G Zhu L Tian Y Song Y. GLS1-mediated glutaminolysis unbridled by MALT1 protease promotes psoriasis pathogenesis. J Clin Invest. (2020) 130:5180–96. doi: 10.1172/JCI129269 PMC7524468 32831293

[161] Edwards DN Ngwa VM Raybuck AL Wang S Hwang Y Kim LC. Selective glutamine metabolism inhibition in tumor cells improves antitumor T lymphocyte activity in triple-negative breast cancer. J Clin Invest. (2021) 131(4):e140100. doi: 10.1172/JCI140100 33320840 PMC7880417

[162] Nitz K Lacy M Bianchini M Wichapong K Kücükgöze IA Bonfiglio CA. The amino acid homoarginine inhibits atherogenesis by modulating T-cell function. Circ Res. (2022) 131:701–12. doi: 10.1161/CIRCRESAHA.122.321094 36102188

[163] Angajala A Lim S Phillips JB Kim JH Yates C You Z. Diverse roles of mitochondria in immune responses: novel insights into immuno-metabolism. Front Immunol. (2018) 9:1605. doi: 10.3389/fimmu.2018.01605 30050539 PMC6052888

[164] Kwong E Li Y Hylemon PB Zhou H. Bile acids and sphingosine-1-phosphate receptor 2 in hepatic lipid metabolism. Acta Pharm Sin B. (2015) 5:151–7. doi: 10.1016/j.apsb.2014.12.009 PMC4629213 26579441

[165] Hang S Paik D Yao L Kim E Trinath J Lu J. Bile acid metabolites control T(H)17 and T(reg) cell differentiation. Nature. (2019) 576:143–8. doi: 10.1038/s41586-019-1785-z PMC6949019 31776512

[166] Cong J Liu P Han Z Ying W Li C Yang Y. Bile acids modified by the intestinal microbiota promote colorectal cancer growth by suppressing CD8(+) T cell effector functions. Immunity. (2024) 57:876–89.e11. doi: 10.1016/j.immuni.2024.02.014 38479384

[167] Song X Sun X Oh SF Wu M Zhang Y Zheng W. Microbial bile acid metabolites modulate gut RORγ(+) regulatory T cell homeostasis. Nature. (2020) 577:410–5. doi: 10.1038/s41586-019-1865-0 PMC7274525 31875848

[168] Haring E Uhl FM Andrieux G Proietti M Bulashevska A Sauer B. Bile acids regulate intestinal antigen presentation and reduce graft-versus-host disease without impairing the graft-versus-leukemia effect. Haematologica. (2021) 106:2131–46. doi: 10.3324/haematol.2019.242990 PMC8327708 32675222

[169] Michonneau D Latis E Curis E Dubouchet L Ramamoorthy S Ingram B. Metabolomics analysis of human acute graft-versus-host disease reveals changes in host and microbiota-derived metabolites. Nat Commun. (2019) 10:5695. doi: 10.1038/s41467-019-13498-3 31836702 PMC6910937

[170] Ding C Hong Y Che Y He T Wang Y Zhang S. Bile acid restrained T cell activation explains cholestasis aggravated hepatitis B virus infection. FASEB J. (2022) 36:e22468. doi: 10.1096/fj.202200332R 35913801

[171] Cao W Kayama H Chen ML Delmas A Sun A Kim SY. The xenobiotic transporter mdr1 enforces T cell homeostasis in the presence of intestinal bile acids. Immunity. (2017) 47:1182–96.e10. doi: 10.1016/j.immuni.2017.11.012 29262351 PMC5741099

[172] Pols TWH Puchner T Korkmaz HI Vos M Soeters MR de Vries CJM. Lithocholic acid controls adaptive immune responses by inhibition of Th1 activation through the Vitamin D receptor. PloS One. (2017) 12:e0176715. doi: 10.1371/journal.pone.0176715 28493883 PMC5426628

[173] Li W Hang S Fang Y Bae S Zhang Y Zhang M. A bacterial bile acid metabolite modulates T(reg) activity through the nuclear hormone receptor NR4A1. Cell Host Microbe. (2021) 29:1366–77.e9. doi: 10.1016/j.chom.2021.07.013 34416161 PMC9064000

[174] Xun Z Lin J Yu Q Liu C Huang J Shang H. Taurocholic acid inhibits the response to interferon-α therapy in patients with HBeAg-positive chronic hepatitis B by impairing CD8(+) T and NK cell function. Cell Mol Immunol. (2021) 18:461–71. doi: 10.1038/s41423-020-00601-8 PMC8027018 33432062

[175] Sun H Guo Y Wang H Yin A Hu J Yuan T. Gut commensal Parabacteroides distasonis alleviates inflammatory arthritis. Gut. (2023) 72:1664–77. doi: 10.1136/gutjnl-2022-327756 36604114

[176] Sun R Zhang Z Bao R Guo X Gu Y Yang W. Loss of SIRT5 promotes bile acid-induced immunosuppressive microenvironment and hepatocarcinogenesis. J Hepatol. (2022) 77:453–66. doi: 10.1016/j.jhep.2022.02.030 35292350

[177] Liu J Wei Y Jia W Can C Wang R Yang X. Chenodeoxycholic acid suppresses AML progression through promoting lipid peroxidation via ROS/p38 MAPK/DGAT1 pathway and inhibiting M2 macrophage polarization. Redox Biol. (2022) 56:102452. doi: 10.1016/j.redox.2022.102452 36084349 PMC9465103

[178] Pi Y Wu Y Zhang X Lu D Han D Zhao J. Gut microbiota-derived ursodeoxycholic acid alleviates low birth weight-induced colonic inflammation by enhancing M2 macrophage polarization. Microbiome. (2023) 11:19. doi: 10.1186/s40168-022-01458-x 36721210 PMC9887892

[179] Wammers M Schupp AK Bode JG Ehlting C Wolf S Deenen R. Reprogramming of pro-inflammatory human macrophages to an anti-inflammatory phenotype by bile acids. Sci Rep. (2018) 8:255. doi: 10.1038/s41598-017-18305-x 29321478 PMC5762890

[180] Hao H Cao L Jiang C Che Y Zhang S Takahashi S. Farnesoid X receptor regulation of the NLRP3 inflammasome underlies cholestasis-associated sepsis. Cell Metab. (2017) 25:856–67.e5. doi: 10.1016/j.cmet.2017.03.007 28380377 PMC6624427

[181] Gitto S Falcini M Marra F. Metabolic disorders after liver transplantation. Metab Syndr Relat Disord. (2021) 19:65–9. doi: 10.1089/met.2020.0068 33104408

[182] Anastácio LR Lima AS Toulson Davisson Correia MI. Metabolic syndrome and its components after liver transplantation: incidence, prevalence, risk factors, and implications. Clin Nutr. (2010) 29:175–9. doi: 10.1016/j.clnu.2009.08.008 19783330

[183] Sponga S Vendramin I Ferrara V Marinoni M Valdi G Di Nora C. Metabolic syndrome and heart transplantation: an underestimated risk factor? Transpl Int. (2024) 37:11075. doi: 10.3389/ti.2024.11075 38525207 PMC10959251

[184] Rahim Z Malik K Sarwar S Salim A. Post liver transplant metabolic syndrome: Frequency, predictors and outcome. Pak J Med Sci. (2025) 41:531–5. doi: 10.12669/pjms.41.2.10774 PMC11803791 39926670

[185] Choudhury A Singh SP Desmukh A Sahoo B Eslam M. Post-liver transplant metabolic syndrome. J Clin Exp Hepatol. (2024) 14:101368. doi: 10.1016/j.jceh.2024.101368 38523736 PMC10960134

[186] Prasad GV Huang M Silver SA Al-Lawati AI Rapi L Nash MM. Metabolic syndrome definitions and components in predicting major adverse cardiovascular events after kidney transplantation. Transpl Int. (2015) 28:79–88. doi: 10.1111/tri.12450 25207680

[187] Som R Morris PJ Knight SR. Graft vessel disease following heart transplantation: a systematic review of the role of statin therapy. World J Surg. (2014) 38:2324–34. doi: 10.1007/s00268-014-2543-x 24700094

[188] Fatourou EM Tsochatzis EA. Management of metabolic syndrome and cardiovascular risk after liver transplantation. Lancet Gastroenterol Hepatol. (2019) 4:731–41. doi: 10.1016/S2468-1253(19)30181-5 31387736

[189] Sun L Clarke R Bennett D Guo Y Walters RG Hill M. Causal associations of blood lipids with risk of ischemic stroke and intracerebral hemorrhage in Chinese adults. Nat Med. (2019) 25:569–74. doi: 10.1038/s41591-019-0366-x PMC6795549 30858617

[190] Marfella R Amarelli C Cacciatore F Balestrieri ML Mansueto G D’Onofrio N. Lipid accumulation in hearts transplanted from nondiabetic donors to diabetic recipients. J Am Coll Cardiol. (2020) 75:1249–62. doi: 10.1016/j.jacc.2020.01.018 32192650

[191] Win TS Crisler WJ Dyring-Andersen B Lopdrup R Teague JE Zhan Q. Immunoregulatory and lipid presentation pathways are upregulated in human face transplant rejection. J Clin Invest. (2021) 131(8):e135166. doi: 10.1172/JCI135166 33667197 PMC8262560

[192] Galindo RJ Wallia A. Hyperglycemia and diabetes mellitus following organ transplantation. Curr Diabetes Rep. (2016) 16:14. doi: 10.1007/s11892-015-0707-1 26803650

[193] Stoumpos S Jardine AG Mark PB. Cardiovascular morbidity and mortality after kidney transplantation. Transpl Int. (2015) 28:10–21. doi: 10.1111/tri.12413 25081992

[194] El Aggan H Mahmoud S El Shair H Elabd H. Increased macrophage activation marker soluble CD163 is associated with graft dysfunction and metabolic derangements in renal transplant recipients. BioMed J. (2021) 44:S179–s89. doi: 10.1016/j.bj.2020.09.004 PMC9068521 35300946

[195] Kuang W Raven LM Muir CA. Early post-transplant hyperglycemia and post-transplant diabetes mellitus following heart transplantation. Expert Rev Endocrinol Metab. (2024) 19:129–40. doi: 10.1080/17446651.2024.2307011 38251642

[196] Prasad GVR Nash MM Yuan W Beriault D Yazdanpanah M Connelly PW. Plasma branched-chain amino acid concentrations and glucose homeostasis in kidney transplant recipients and candidates. Can J Kidney Health Dis. (2023) 10:20543581231168085. doi: 10.1177/20543581231168085 37101847 PMC10123875

[197] Cheng X Ge M Zhu S Li D Wang R Xu Q. mTORC1-mediated amino acid signaling is critical for cell fate determination under transplant-induced stress. FEBS Lett. (2021) 595:462–75. doi: 10.1002/1873-3468.14008 33249578

[198] Weischendorff S Kielsen K Nederby M Schmidt L Burrin D Heilmann C. Reduced plasma amino acid levels during allogeneic hematopoietic stem cell transplantation are associated with systemic inflammation and treatment-related complications. Biol Blood Marrow Transplant. (2019) 25:1432–40. doi: 10.1016/j.bbmt.2019.03.018 30910606

[199] Hammad A Kaido T Aliyev V Mandato C Uemoto S. Nutritional therapy in liver transplantation. Nutrients. (2017) 9(10):1126. doi: 10.3390/nu9101126 29035319 PMC5691742

[200] Redman JS Kaspar M Puri P. Implications of pre-transplant sarcopenia and frailty in patients with non-alcoholic steatohepatitis and alcoholic liver disease. Transl Gastroenterol Hepatol. (2022) 7:29. doi: 10.21037/tgh-20-236 35892054 PMC9257536

[201] Beraza N Ofner-Ziegenfuss L Ehedego H Boekschoten M Bischoff SC Mueller M. Nor-ursodeoxycholic acid reverses hepatocyte-specific nemo-dependent steatohepatitis. Gut. (2011) 60:387–96. doi: 10.1136/gut.2010.223834 21115542

[202] Lehmann CJ Dylla NP Odenwald M Nayak R Khalid M Boissiere J. Fecal metabolite profiling identifies liver transplant recipients at risk for postoperative infection. Cell Host Microbe. (2024) 32:117–30.e4. doi: 10.1016/j.chom.2023.11.016 38103544

[203] van Raalte DH Ouwens DM Diamant M. Novel insights into glucocorticoid-mediated diabetogenic effects: towards expansion of therapeutic options? Eur J Clin Invest. (2009) 39:81–93. doi: 10.1111/j.1365-2362.2008.02067.x 19200161

[204] Knight SR Morris PJ. Steroid avoidance or withdrawal after renal transplantation increases the risk of acute rejection but decreases cardiovascular risk. A meta-analysis. Transplantation. (2010) 89:1–14. doi: 10.1097/TP.0b013e3181c518cc 20061913

[205] Hoogeveen RC Ballantyne CM Pownall HJ Opekun AR Hachey DL Jaffe JS. Effect of sirolimus on the metabolism of apoB100- containing lipoproteins in renal transplant patients. Transplantation. (2001) 72:1244–50. doi: 10.1097/00007890-200110150-00011 11602850

[206] Johnston O Rose CL Webster AC Gill JS. Sirolimus is associated with new-onset diabetes in kidney transplant recipients. J Am Soc Nephrol. (2008) 19:1411–8. doi: 10.1681/ASN.2007111202 PMC2440303 18385422

[207] Roland M Gatault P Doute C Büchler M Al-Najjar A Barbet C. Immunosuppressive medications, clinical and metabolic parameters in new-onset diabetes mellitus after kidney transplantation. Transpl Int. (2008) 21:523–30. doi: 10.1111/j.1432-2277.2008.00640.x 18266773

[208] Teutonico A Schena PF Di Paolo S. Glucose metabolism in renal transplant recipients: effect of calcineurin inhibitor withdrawal and conversion to sirolimus. J Am Soc Nephrol. (2005) 16:3128–35. doi: 10.1681/ASN.2005050487 16107580

[209] McCune TR Thacker LR II Peters TG Mulloy L Rohr MS Adams PA. Effects of tacrolimus on hyperlipidemia after successful renal transplantation: a Southeastern Organ Procurement Foundation multicenter clinical study. Transplantation. (1998) 65:87–92. doi: 10.1097/00007890-199801150-00017 9448150

[210] Tamura K Fujimura T Tsutsumi T Nakamura K Ogawa T Atumaru C. Transcriptional inhibition of insulin by FK506 and possible involvement of FK506 binding protein-12 in pancreatic beta-cell. Transplantation. (1995) 59:1606–13. 7539960

[211] Filler G Neuschulz I Vollmer I Amendt P Hocher B. Tacrolimus reversibly reduces insulin secretion in paediatric renal transplant recipients. Nephrol Dial Transplant. (2000) 15:867–71. doi: 10.1093/ndt/15.6.867 10831643

[212] Haller MC Royuela A Nagler EV Pascual J Webster AC. Steroid avoidance or withdrawal for kidney transplant recipients. Cochrane Database Syst Rev. (2016) 2016:Cd005632. doi: 10.1002/14651858.CD005632.pub3 27546100 PMC8520739

[213] Sharif A Chakkera H de Vries APJ Eller K Guthoff M Haller MC. International consensus on post-transplantation diabetes mellitus. Nephrol Dial Transplant. (2024) 39:531–49. doi: 10.1093/ndt/gfad258 PMC11024828 38171510

[214] Montero N Quero M Melilli E Pérez-Sáez MJ Redondo-Pachón D Bestard O. Mammalian target of rapamycin inhibitors combined with calcineurin inhibitors as initial immunosuppression in renal transplantation: A meta-analysis. Transplantation. (2019) 103:2031–56. doi: 10.1097/TP.0000000000002769 31343574

[215] Neal DA Gimson AE Gibbs P Alexander GJ. Beneficial effects of converting liver transplant recipients from cyclosporine to tacrolimus on blood pressure, serum lipids, and weight. Liver Transpl. (2001) 7:533–9. doi: 10.1053/jlts.2001.24637 11443583

[216] Grundy SM Cleeman JI Daniels SR Donato KA Eckel RH Franklin BA. Diagnosis and management of the metabolic syndrome: an American Heart Association/National Heart, Lung, and Blood Institute Scientific Statement. Circulation. (2005) 112:2735–52. doi: 10.1161/CIRCULATIONAHA.105.169404 16157765

[217] Castro-Barquero S Ruiz-León AM Sierra-Pérez M Estruch R Casas R. Dietary strategies for metabolic syndrome: A comprehensive review. Nutrients. (2020) 12(10):2983 doi: 10.3390/nu12102983 33003472 PMC7600579

[218] Fahed G Aoun L Bou Zerdan M Allam S Bou Zerdan M Bouferraa Y. Metabolic syndrome: updates on pathophysiology and management in 2021. Int J Mol Sci. (2022) 23(2):786. doi: 10.3390/ijms23020786 35054972 PMC8775991

[219] Pandya V Rao A Chaudhary K. Lipid abnormalities in kidney disease and management strategies. World J Nephrol. (2015) 4:83–91. doi: 10.5527/wjn.v4.i1.83 25664249 PMC4317631

[220] Didsbury M McGee RG Tong A Craig JC Chapman JR Chadban S. Exercise training in solid organ transplant recipients: a systematic review and meta-analysis. Transplantation. (2013) 95:679–87. doi: 10.1097/TP.0b013e31827a3d3e 23364480

[221] Abbaszadeh S Nosrati-Siahmazgi V Musaie K Rezaei S Qahremani M Xiao B. Emerging strategies to bypass transplant rejection via biomaterial-assisted immunoengineering: Insights from islets and beyond. Adv Drug Delivery Rev. (2023) 200:115050. doi: 10.1016/j.addr.2023.115050 37549847

[222] Pérez-Martínez P Mikhailidis DP Athyros VG Bullo M Couture P Covas MI. Lifestyle recommendations for the prevention and management of metabolic syndrome: an international panel recommendation. Nutr Rev. (2017) 75:307–26. doi: 10.1093/nutrit/nux014 PMC5914407 28521334

[223] Chowdhury TA Wahba M Mallik R Peracha J Patel D De P. Association of British Clinical Diabetologists and Renal Association guidelines on the detection and management of diabetes post solid organ transplantation. Diabetes Med. (2021) 38:e14523. doi: 10.1111/dme.14523 33434362

[224] Hecking M Sharif A Eller K Jenssen T. Management of post-transplant diabetes: immunosuppression, early prevention, and novel antidiabetics. Transpl Int. (2021) 34:27–48. doi: 10.1111/tri.13783 33135259 PMC7839745

[225] Tonelli M Wanner C. Lipid management in chronic kidney disease: synopsis of the Kidney Disease: Improving Global Outcomes 2013 clinical practice guideline. Ann Intern Med. (2014) 160:182. doi: 10.7326/M13-2453 24323134

[226] Melendez QM Krishnaji ST Wooten CJ Lopez D. Hypercholesterolemia: the role of PCSK9. Arch Biochem Biophys. (2017) 625-626:39–53. doi: 10.1016/j.abb.2017.06.001 28587771

